# Colon-targeted oral delivery of melatonin for regulating macrophage activity and intestinal barrier in ulcerative colitis

**DOI:** 10.7150/thno.133456

**Published:** 2026-08-24

**Authors:** Nhu-Nam Nguyen, Yoojin Seo, Tiep Tien Nguyen, Kim-Phuong Phan, Rabyya Kausar, Hu-Lin Jiang, Jae Young Lee, Seho Kweon, Nguyen-Thach Tung, Hyung-Sik Kim, Jee-Heon Jeong

**Affiliations:** 1 Department of Precision Medicine, School of Medicine, Sungkyunkwan University, Suwon, Gyeonggi, Republic of Korea.; 2 Department of Oral Biochemistry, Dental and Life Science Institute, School of Dentistry, Pusan National University, Yangsan, Republic of Korea.; 3 State Key Laboratory of Natural Medicines, Department of Pharmaceutics, China Pharmaceutical University, Nanjing, China.; 4 College of Pharmacy and Research Institute of Pharmaceutical Sciences, Seoul National University, Seoul, Republic of Korea.; 5 College of Pharmacy, Chonnam National University, Gwangju, Republic of Korea.; 6 Department of Pharmaceutics, Hanoi University of Pharmacy, Vietnam.

**Keywords:** ulcerative colitis, melatonin, colon-targeted oral delivery, macrophage modulation, intestinal barrier

## Abstract

**Background:**

Despite its promising therapeutic potential, inefficient site-specific targeting and complex fabrication processes hinder the clinical translation of oral melatonin therapy for ulcerative colitis (UC). Herein, we present **M**elatonin-**E**udragit-**T**hioketal polymer-based **A**ssembly (**META**), a dual pH/ROS-responsive oral microsphere for precise melatonin delivery to the inflamed colon and enhanced therapeutic outcomes.

**Methods:**

META was fabricated via a scalable emulsification process using Eudragit^®^ FS 30 D and a thioketal polymer to enable dual pH- and ROS-responsive release. Its targeting delivery and therapeutic effects were then evaluated in simulated gastrointestinal (GI) fluids, 2D cell models, intestinal organoids, and a murine colitis model.

**Results:**

Using cell and animal models, we showed the important role of melatonin in GI health, supporting our strategy for UC treatment. META exhibited controlled melatonin release and better accumulation in the inflamed colon. The targeted delivery helped promote disease recovery by modulating macrophages and improving the intestinal barrier.

**Conclusion:**

META delivers melatonin precisely to the colon, which reprograms colonic macrophages and reinforces the epithelial barrier. This study offers a practical, clinically relevant approach for improving the use of endogenous therapeutic molecules in UC and other inflammatory GI diseases.

## Introduction

Ulcerative colitis (UC) is a chronic inflammatory bowel disease (IBD) that mainly affects the large intestine and leads to persistent inflammation and ulceration of the colonic mucosa 1, 2. Its incidence has continued to rise worldwide, making it an increasing long-term burden for millions of people 2-4. The disease develops through a complex interplay of genetic susceptibility, impaired epithelial barrier function, dysregulated immune responses, and environmental factors 5, 6. Macrophages are central to this process. They arise from circulating monocytes and respond to inflammatory signals by differentiating into cells 7 that help maintain intestinal homeostasis. In UC, however, this balance is disrupted, and a shift toward pro-inflammatory M1 macrophages rather than anti-inflammatory M2 macrophages is thought to drive disease progression 8, 9. Current treatment options mainly include anti-inflammatory drugs, immunomodulators, and biologics such as 5-aminosalicylic acid (5-ASA) agents, corticosteroids, immunosuppressants, and anti-TNF-α therapies 1, 2. Although these treatments can help control symptoms, they often act on only one part of the inflammatory process rather than the whole disease mechanism. As a result, many patients do not achieve complete remission, and systemic side effects remain a serious concern 10, 11. Immunosuppressive drugs can weaken normal immune function and increase the risk of infection and organ toxicity, while biologics are often limited by poor stability, short half-life, high cost, and the need for repeated intravenous administration, which can reduce compliance and overall treatment effectiveness 10, 12, 13. Together, these limitations highlight the need for safer and more effective therapies with better delivery systems 14, 15.

Melatonin (*N*-acetyl-5-methoxytryptamine) is best known for its role in regulating circadian rhythms, but it also acts as a potent immunomodulator and antioxidant 16. In addition to being secreted by the pineal gland, melatonin is produced locally by enterochromaffin cells in the gastrointestinal (GI) tract 17, 18. It exerts antioxidant and anti-inflammatory effects that may be beneficial in a range of GI disorders 19, 20. As an endogenous molecule, melatonin has the advantage of good biocompatibility, which may improve both efficacy and tolerability compared with conventional therapeutic agents 21. Although preclinical and clinical studies have suggested that melatonin can reduce intestinal damage and alleviate UC symptoms, its therapeutic potential remains largely underexplored 22-25. Because melatonin is stable in the acidic environment of the stomach 26, it has been considered for oral delivery, a route favored by patients for its convenience and potential access to the colon 19. However, oral melatonin delivery is still limited by poor pharmacokinetics, insufficient accumulation in inflamed colonic tissue, limited retention at disease sites, and the hostile inflammatory microenvironment that reduces local drug activity 27-30. In many cases, current formulations are absorbed too early in the small intestine, resulting in poor colonic accumulation and unwanted systemic effects 31, 32. Advanced oral delivery systems for melatonin in UC have not yet reached clinical trials, and most studies still rely on free melatonin (Table **S1**). This gap underscores the need for precisely responsive delivery systems tailored to the unique pathophysiology of UC to improve therapeutic outcomes.

Although many advanced oral delivery platforms have been developed for UC, poor targeting to diseased tissue and complicated fabrication processes continue to limit their clinical translation 33-36. For melatonin, a simple but effective platform that improves site-specific delivery could help unlock its full therapeutic potential and support clinical use. An effective oral delivery system for melatonin should consider disease-related changes in GI physiology, including altered pH and elevated reactive oxygen species (ROS) levels, to achieve precise targeting and consistent therapeutic effects 37, 38. ROS levels in intestinal lesions from patients with IBD have been reported to be 10- to 100-fold higher than those in healthy individuals and play an important role in colitis progression 38, 39. For this reason, ROS-responsive delivery systems are considered promising for UC treatment 39-45. However, the spatial variability of ROS along the GI tract raises the risks of premature or off-target activation, which can reduce reliability 40, 46. Similarly, the distinct pH profile along the GI tract has inspired many colon-targeted formulations based on pH-dependent release 47-49. Yet variation in luminal pH among patients, as well as differences between healthy and diseased states, can reduce the consistency and effectiveness of these systems 43, 50, 51. Given these challenges, monotherapies based on either ROS or pH responsiveness alone are often not sufficient, especially for melatonin, which requires highly precise delivery. By combining both triggers, a synergistic pH/ROS-responsive strategy can better navigate the dynamic GI microenvironment, allowing more controlled oral release of melatonin and improving therapeutic outcomes in UC.

**Figure 1:**
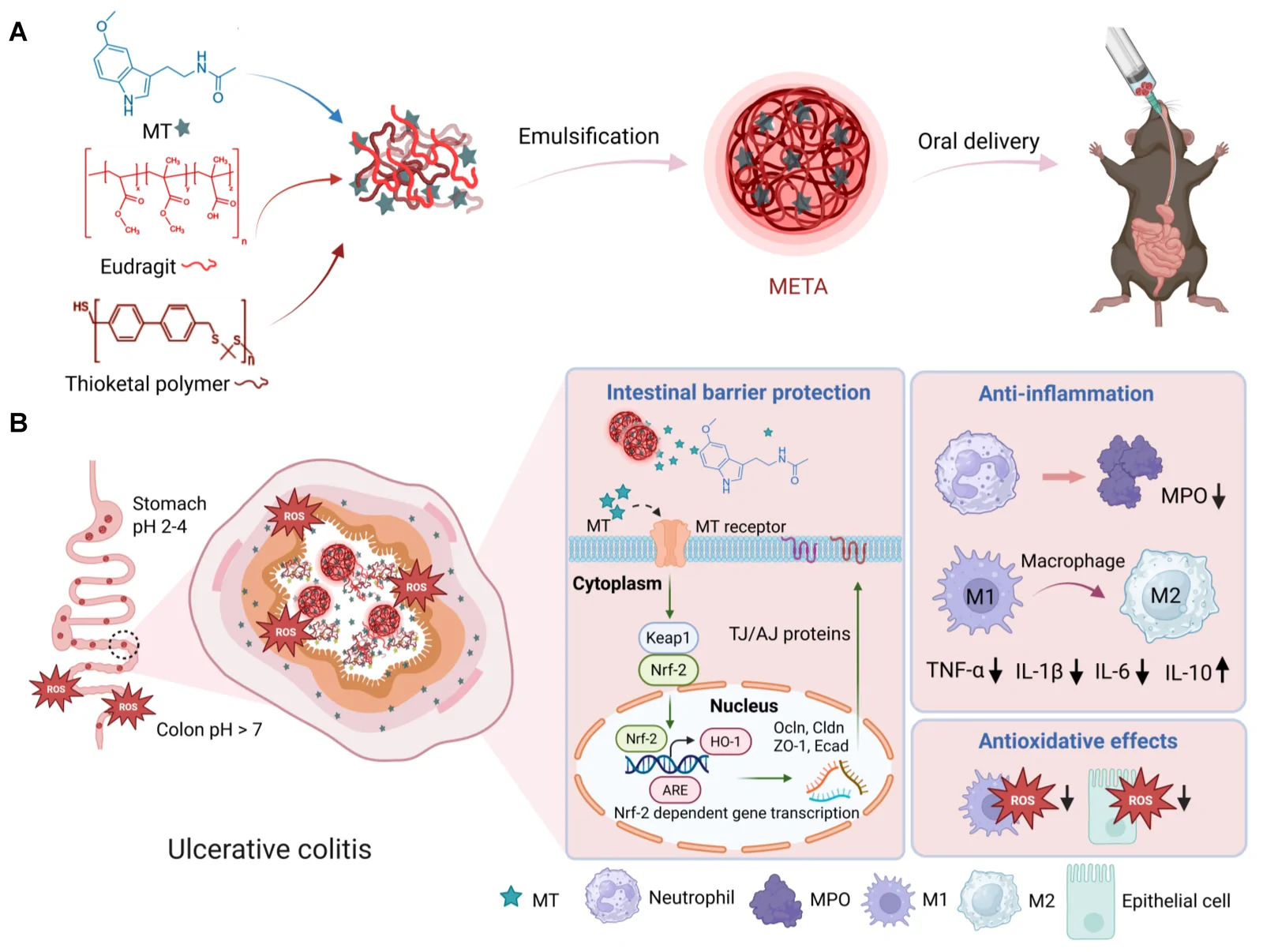
** Schematic illustration of META preparation and its mechanism of action in UC treatment. A,** META fabrication and administration. **B,** Melatonin is released and accumulates specifically at injured colon sites, where it exerts therapeutic effects by modulating macrophage polarization and enhancing intestinal barrier integrity. MT: melatonin, TJ: tight junction, AJ: adherens junction.

In this study, we developed an oral delivery system for melatonin, **M**elatonin-**E**udragit-**T**hioketal polymer-based **A**ssembly (**META**), designed to respond to both pH and ROS. By taking advantage of these two disease-related triggers, the system can more selectively target inflamed colonic tissue and improve therapeutic efficacy in a murine colitis model. META incorporates biocompatible and functional materials, including Eudragit^®^ FS 30 D, an FDA-approved excipient that responds to high pH 37, 52, and thioketal polymers that are sensitive to elevated ROS levels 43. These components form a polymeric matrix that encapsulates melatonin through a simple and scalable emulsification process (Figure **1**). Compared with more complex delivery platforms, this approach is easier to prepare and may be more practical for future translation. Beyond developing a delivery system, we also sought to better understand the role of melatonin in GI physiology and its mechanism in colonic repair via macrophage modulation and intestinal barrier reinforcement. Using single-cell analysis, intestinal organoid models, and *in vivo* colitis mouse models, we showed that META achieved effective colonic targeting and meaningful therapeutic benefit, with macrophages playing an important role in the response. Collectively, these findings may advance the understanding of melatonin-based therapies and offer a promising, clinically translatable strategy for treating UC.

## Results

### Relationship between melatonin and colonic inflammation

**Figure 2:**
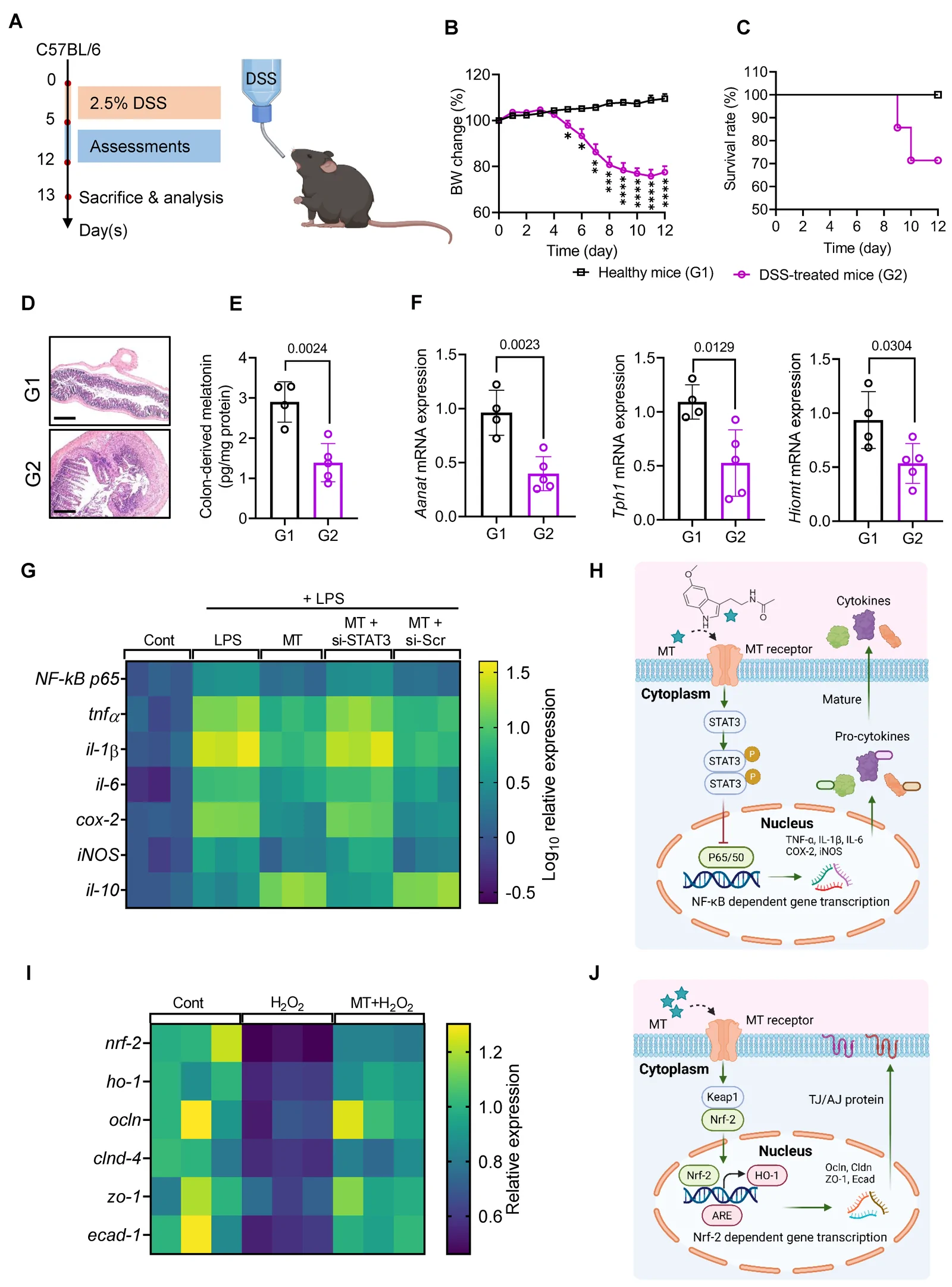
** Relationship between melatonin and colonic inflammation. A,** Induction of the mouse colitis model through oral dextran sulfate sodium (DSS) treatment. **B,** Body weight change measurement (n = 4-7 mice). **C,** Survival rate of mice (n = 4-7 mice).** D,** Representative histological images of colon tissue were obtained with H&E staining. Scale bar, 250 µm. **E,** Quantification of melatonin levels in colon tissue by ELISA (n = 4-5 mice). **F,** Relative mRNA expression of *Aanat, Tph1,* and *Hiomt* derived from the colon tissue using RT-PCR (n = 4-5 mice). **G,** Heat map depicting mRNA expression in RAW 264.7 macrophages (n = 3). **H,** Melatonin’s mechanism of action underlying macrophage modulation. **I,** Heat map depicting mRNA expression in Caco-2 (n = 3). **J,** Melatonin’s mechanism of action underlying protective effects. Data in (**B**) are presented as mean ± s.e.m. Data in (**E, F**) are presented as mean ± s.d. Data in (**B, E, F**) are analyzed using an unpaired two-tailed t-test. In (**B**) ^*^p < 0.05, ^**^p < 0.01, ^***^p < 0.001, ^****^p < 0.0001. In (**E, F**), significant p values are shown. Figures **2A, H, J** were created with BioRender.com. MT: melatonin, TJ: tight junction, AJ: adherens junction, LPS: lipopolysaccharide, si-Scr: scrambled negative control siRNA.

Melatonin (MT), which is abundantly secreted by enterochromaffin cells in the GI tract, plays an important role in maintaining gut integrity 53. To investigate whether melatonin is involved in UC, we established a dextran sulfate sodium (DSS)-induced colitis model in C57BL/6 mice (Figure **2A**). The model closely reproduces the major clinical and histological features of human UC 54. Compared with healthy mice, DSS-treated mice showed marked body weight loss, reduced survival, and higher disease activity index (DAI) scores, indicating more severe disease (Figures **2B, C,** and Figure** S1**). Histological examination also revealed crypt damage, epithelial injury, and inflammatory cell infiltration (Figure **2D**). We then evaluated melatonin levels in the colon of both healthy and diseased mice. Mice with colitis showed significantly lower colonic melatonin and downregulated melatonin biosynthetic genes such as *Aanat*, *Tph1*, and *Hiomt* (Figure **2E, F**). Taken together, these results suggest an association between lowered melatonin levels and intestinal damage in UC, and they also imply that melatonin deficiency may drive disease progression.

Based on the known anti-inflammatory and cytoprotective effects of melatonin in intestinal disorders, we further examined its impact on macrophage polarization, which is a key process in UC pathogenesis. Because STAT3 is an important regulator of inflammation 55, we used siRNA to knock down STAT3 in RAW 264.7 macrophages, with a scrambled negative control siRNA (si-Scr), to determine whether STAT3 signaling is required for melatonin’s anti-inflammatory effects. We confirmed efficient suppression of STAT3 at both the mRNA and protein levels (Figure **S2A, B**). Lipopolysaccharide (LPS) stimulation promoted M1 polarization, as shown by increased NF-κB p65 expression and upregulation of inflammatory genes. In contrast, melatonin treatment strongly suppressed these responses while increasing IL-10 expression, a marker associated with M2 macrophages (Figure **2G**). Importantly, STAT3 silencing reduced these anti-inflammatory effects of melatonin, indicating that STAT3 signaling is necessary for melatonin activity in RAW 264.7 cells (Figures **2G, H**). Melatonin increased p-STAT3 expression in a concentration-dependent manner (Figure **S2C**), suggesting activation of the STAT3 pathway. In addition, the STAT3 inhibitor stattic weakened the effects of melatonin on macrophage polarization (Figure **S2D-G**). Taken together, these findings suggest that melatonin regulates macrophage polarization largely through STAT3 signaling.

Melatonin also protected Caco-2 colonic epithelial cells from H_2_O_2_-induced injury and increased tight and adherens junction factors (Figure **2I**). Mechanistic analysis suggested that this protective effect was mediated through the NRF-2/HO-1 pathway (Figures **2I, J**). In addition, melatonin showed clear antioxidant activity (Figure **S3**), further supporting its ability to counter colitis-like stress. Together, these findings highlight the important role of melatonin in UC and suggest that melatonin supplementation may be a promising therapeutic option for UC.

### Fabrication and characterization of META

We developed a dual pH- and ROS-responsive microsphere platform, namely **META**, designed to address the limitations of previous systems and precisely deliver melatonin to colonic inflammation sites via synergistic responsiveness.

**Figure 3:**
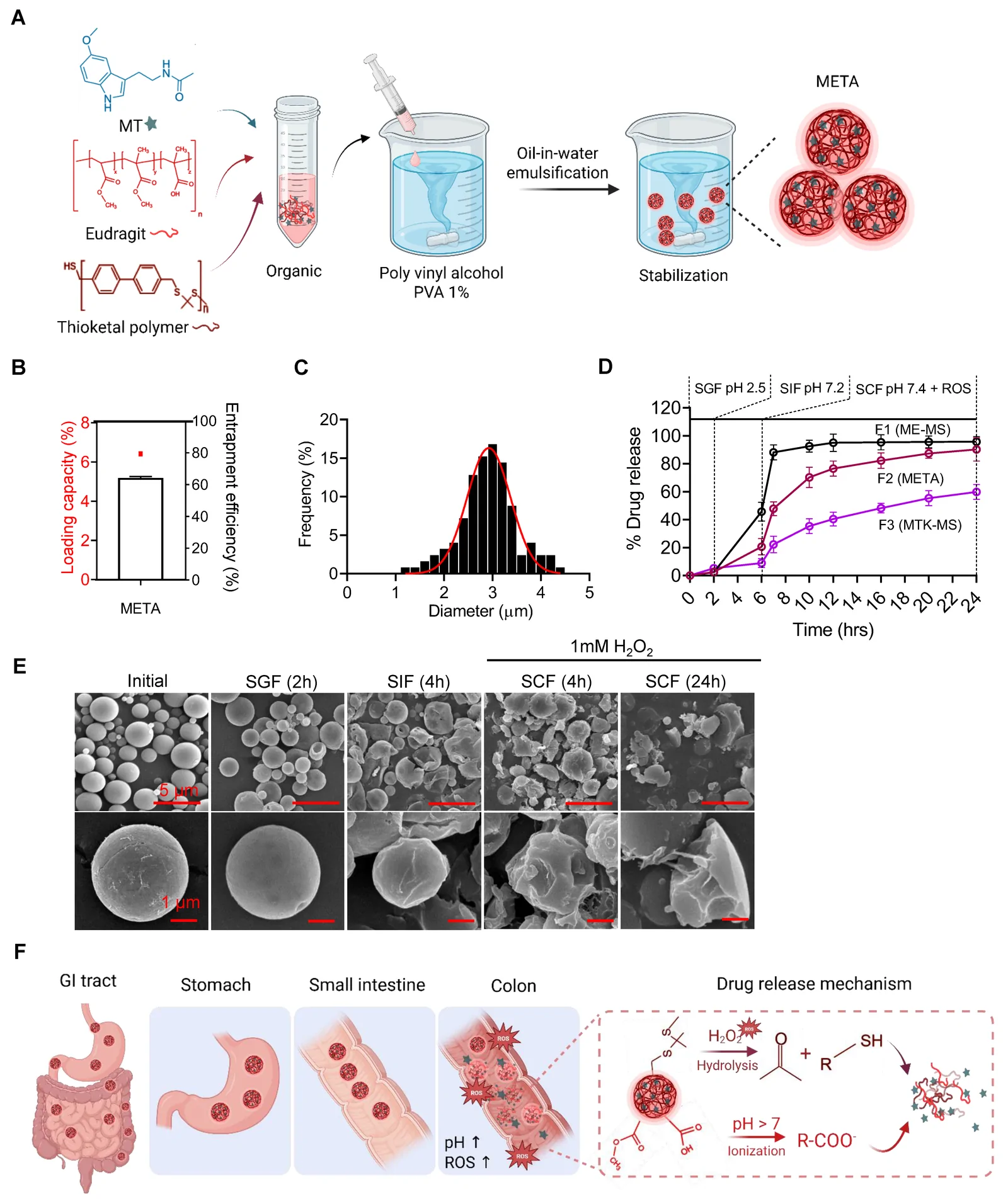
** Fabrication and characterization of META. A,** Fabrication of the META platform. **B,** Melatonin loading capacity and entrapment efficiency of META (n = 3). **C,** META size distribution. **D,** Melatonin release profile of melatonin-loaded microspheres (n = 3). **E,** SEM images of META in GI tract-mimicking conditions in UC. **F,** Drug release mechanism of META in the GI tract. Figures **3A, F** were created with BioRender.com. MT: melatonin, PVA: polyvinyl alcohol, SGF: simulated gastric fluid, SIF: simulated intestinal fluid, SCF: simulated colonic fluid.

META was fabricated by a simple and scalable emulsification method as illustrated in Figure **3A**. Eudragit^®^ FS 30 D is an FDA-approved excipient for drug development with high pH-responsive properties (above pH 7) 37, 52. The thioketal polymer was developed with highly biocompatible properties and introduced in our previous study 43. The confirmed chemical structure showed that the ROS-sensitive linker enables this polymer to respond effectively in an enriched ROS environment (Figure** S4**). Individual polymer-based microspheres were also prepared and characterized following the same method for comparative purposes (Figure **S5A-F**). The melatonin loading capacity and entrapment efficiency of META were 6.41 ± 0.09% and 64.15 ± 0.91%, respectively (Figure **3B**). META exhibited a spherical shape with a uniform size of approximately 3 µm (Figures **3C, E-**initial timepoint) and a negative zeta potential, indicating favorable dispersibility and colloidal stability (Figure **S5D, G**). To better mimic disease-relevant conditions, luminal pH was first measured between healthy and colitis mice (Figure **S6**). We then comprehensively evaluated melatonin release from different formulations under simulated gastric fluid (SGF), simulated intestinal fluid (SIF), and simulated colonic fluid (SCF) corresponding to predetermined UC-mimicking conditions. The incubation times were selected to simulate the transit time of a single-unit dosage form in the gastrointestinal tract, specifically 2 h for SGF, 4 h for SIF, and an extended period for SCF 56, 57. Interestingly, META showed minimal release in SGF and SIF but a pronounced, controlled release in SCF with elevated pH and ROS, indicating strong site-specific responsiveness (Figure **3D**). Consistently, SEM images also revealed stimulus-triggered degradation of META with significant morphology changes in different conditions, especially in the SCF (Figure **3E**). Besides, Melatonin-Eudragit microsphere (ME-MS) showed rapid melatonin release (with a similarity factor *f_2_* between META and ME-MS of 34.2) from SIF to SCF due to extensive ionization of Eudragit in high pH conditions (Figure **3D**). In contrast, Melatonin-Thioketal microsphere (MTK-MS) showed a delayed release (with a similarity factor *f_2_* between META and MTK-MS of 27.5), reflecting the slower ROS-mediated cleavage of thioketal linkers (Figure **3D**), as observed in a previous study 43. These data indicated that a rational combination of functional polymers, including pH-responsive Eudragit and ROS-responsive thioketal polymer, effectively controls melatonin release and enhances precise delivery to the inflamed colon (Figure **3F**).

### META exhibits anti-inflammatory effects and intestinal barrier protection

After confirming the cytotoxicity profile of the microsphere (Figure **S7**), we evaluated multiple effects of META on single-cell models.

**Figure 4:**
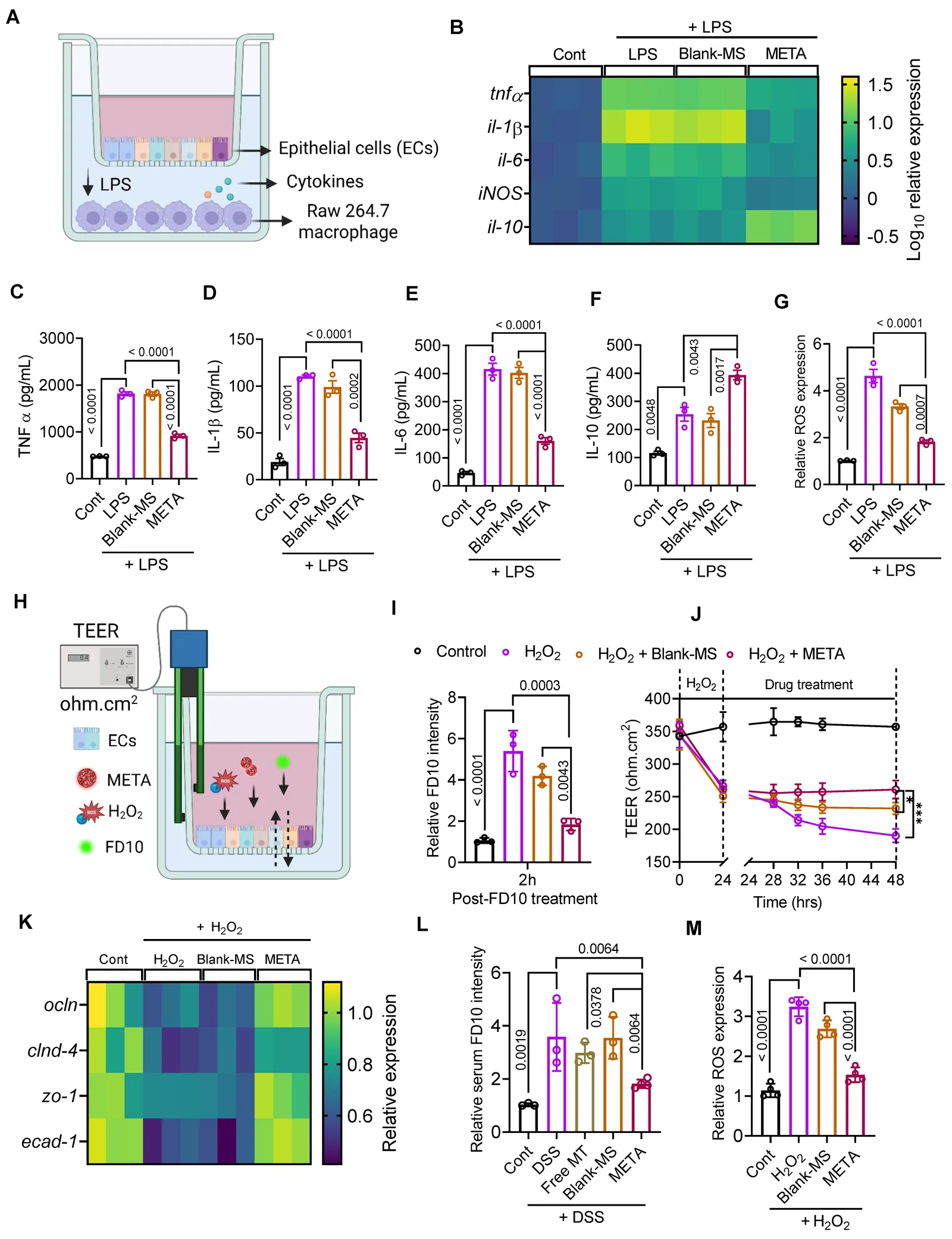
** META exhibits anti-inflammatory effects and protects the intestinal barrier. A,** Co-culture model of the Caco-2 epithelial cells (ECs) and RAW 264.7 macrophages. **B,** Gene expression of inflammatory and anti-inflammatory factors in RAW cells (n = 3). **C-F,** Enzyme-linked immunosorbent assay (ELISA) quantification of extracellular cytokines, including TNF-α, IL-1β, IL-6, and IL-10, respectively (n = 3). **G,** ROS assessments in RAW cells (n = 3). **H,** Epithelial Caco-2 cell monolayer model for *in vitro* permeability assessment. **I,** FD10 intensity measurement in the basolateral chamber (n = 3).** J,** TEER values indicating epithelial barrier integrity, measured between apical and basolateral chambers (n = 3). **K,** Gene expression in the tight and adherens junctions in Caco-2 cells (n = 3). **L,** FD10 intensity measurement in the serum from mice after treatment (n = 3-4 mice). **M,** ROS assessments in Caco-2 cells (n = 4). Data are presented as mean ± s.d. Data are analyzed using one-way ANOVA with Tukey’s multiple comparisons test (significant p values are shown). Figures **4A, H** were created with BioRender.com. TEER: trans-epithelial electrical resistance, FD10: FITC-Dextran 10kDa.

To evaluate the anti-inflammatory effects of META, we used a Caco-2/RAW 264.7 co-culture model (Figure **4A**). RAW 264.7 macrophages were first driven into an inflammatory state with LPS, then treated with different formulations. As expected, inflammatory genes were upregulated in the LPS and Blank-MS groups, but META strongly dampened their expression (Figure **4B**). Additionally, IL-10 mRNA was notably upregulated in RAW cells treated with META compared with the LPS or Blank-MS groups (Figure **4B**). Consistent with these gene changes, cytokine measurements in the conditioned medium showed that META lowered pro-inflammatory cytokines, including TNF-α, IL-1β, and IL-6 (Figures **4C-E**), while boosting IL-10 (Figure **4F**). Altogether, these findings indicate that META suppresses inflammation by decreasing M1 macrophage polarization and promoting M2 activation. Furthermore, META also reduced oxidative stress with reduced ROS levels in treated RAW cells (Figure **4G**), which supports its wider protective profile.

To determine whether META could protect the intestinal barrier from ROS-induced damage, we established a transwell-based epithelial cell model for permeability assessments (Figure **4H** and Figure **S8**). Epithelial disruption was induced by H_2_O_2_ to mimic intestinal injury. META effectively preserved epithelial integrity with a lower FITC-Dextran 10 kDa (FD10) signal in the basolateral chamber compared to the H_2_O_2_ and Blank-MS groups (Figure **4I**). Transepithelial electrical resistance (TEER) measurement further confirmed the protective effects of META, with significantly higher TEER values than in the H_2_O_2_ and Blank-MS groups (Figure **4J**). In addition, META increased the expression of tight junction and adherens junction factors, indicating improved epithelial barrier function (Figure **4K**). Importantly, in a murine colitis model, META showed better protective effects than other groups with lower serum FD10 levels (Figure **4L**). Although free melatonin showed protective effects *in vitro* (Figure **2I, J**), orally delivered melatonin had little impact on the colitis model, underscoring the need for targeted delivery to achieve therapeutic benefit (Figure **4L**). Besides, Caco-2 cells treated with META also showed lower ROS levels, supporting the antioxidant capacity of this promising therapy (Figure **4M**). Together, these results suggest META provides broad protection in colitis by combining antioxidant effects with reinforcement of the intestinal barrier, which may inhibit disease progression.

### META notably preserves intestinal organoid integrity

**Figure 5:**
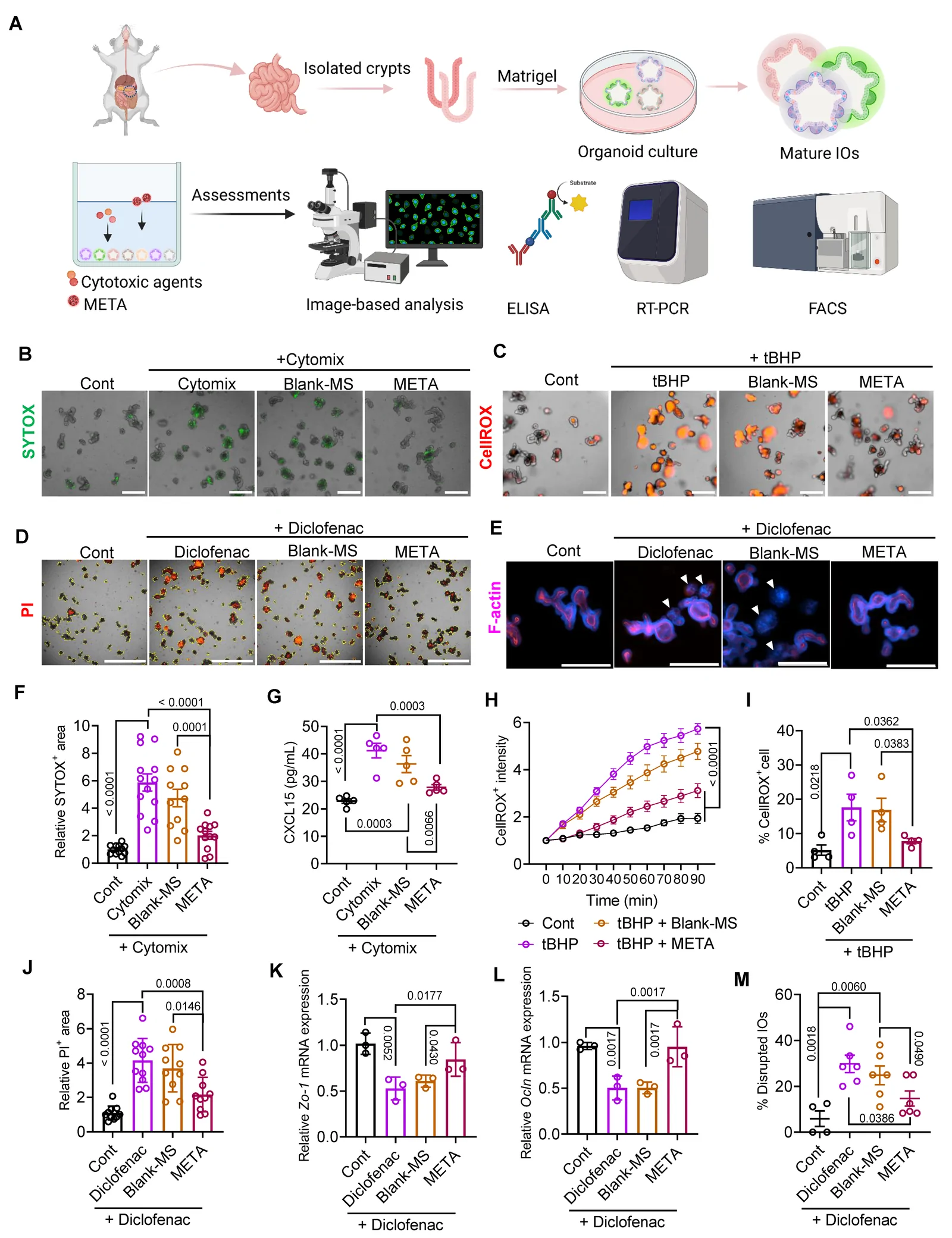
** META notably preserves intestinal organoid integrity. A,** Intestinal organoid (IO)-based assessments are shown. **B,** Representative images of Cytomix-induced IOs damage stained with SYTOX. Scale bar: 300 µm. **C,** CellROX staining showing oxidative stress-induced damage in IOs treated with tBHP. Scale bar: 300 µm. **D,** Propidium iodide (PI) staining depicts diclofenac-induced IO damage. Scale bar: 1000 µm. **E,** F-actin staining shows changes in organoid morphology and epithelial integrity after diclofenac treatment. Arrows indicate the disrupted structure. Scale bar: 300 µm. **F,** SYTOX-positive area of IO measurement (n = 11-12).** G,** CXCL15 concentration secreted from IOs (n = 5). **H,** Time course of CellROX intensity in IOs (n = 3-8). **I,** Percentage of CellROX-positive cells by flow cytometry after 60 min of treatment (n = 4). **J,** PI-positive area of IO measurement (n = 10-11). **K-L,** Relative mRNA expression of *Zo-1* and *Ocln* in IOs (n = 3). **M,** Percentage of disrupted IOs (n = 4-6). In (**F-M**), data are presented as mean ± s.e.m. Data in (**F-M**) are analyzed using one-way ANOVA with the two-stage linear step-up method of Benjamini, Krieger, and Yekutieli (significant q values are shown). Figure **5A** was created with BioRender.com. IOs: intestinal organoids, PI: propidium iodide, SYTOX: dead-cell nucleic acid stain, CellROX: ROS detection dye.

META demonstrated excellent intestinal barrier protection, as evidenced by both the 2D cell model in vitro and the mouse colitis model (Figure **4**). However, to further explore and comprehensively characterize META’s protective therapeutic effects, an intestinal organoid-based model was further employed (Figure **5A**).

Upon confirming that neither META nor Blank-MS adversely affected organoid growth or viability (Figures **S9A, B**), we investigated the protective role of META in intestinal organoids (IOs) challenged with various cytotoxic agents (Figure **5A**). To stimulate inflammation-mediated injury, IOs were exposed to a combination of pro-inflammatory cytokines, including IFN-γ, TNF-α, and IL-1β (Cytomix), for 24 h. Following treatment, the proportion of dead cells (SYTOX^+^) within the organoids increased significantly (Figures **5B, F**). This tendency was mitigated by co-treatment with META in a dose-dependent manner, whereas Blank-MS showed no effect (Figures **5B, F**). META treatment also significantly attenuated the secretion of the important immune mediator CXCL15/IL-8 in IOs in response to cytomix exposure (Figure **5G**), indicating its ability to interrupt the inflammatory cascade in epithelial cells.

Building on the antioxidant effects of META observed in 2D epithelial cell models (Figure **4**), we further examined its protective capacity against oxidative stress in a more physiologically relevant IO model. Real-time imaging showed that intracellular ROS levels rose quickly after tBHP treatment, confirming that oxidative stress had been successfully triggered (Figures **5C, H**, and Figure **S9C**). The Blank-MS group showed similarly high ROS levels, almost the same as the tBHP group, while META treatment significantly attenuated tBHP-induced ROS accumulation (Figures **5C, H**, and Figure **S9C**). Consistent with these findings, flow cytometric analysis further confirmed that META reduced the fraction of ROS-high cells to less than half compared to the tBHP-treated group (Figure **5I** and Figure **S9D**). Overall, these data reinforce that META offers strong protection against ROS-induced damage to the intestinal barrier.

Next, we modeled NSAID-induced enteropathy by treating organoids with diclofenac, which disrupts epithelial tight junctions 58. In the PI staining-based permeability assay, diclofenac-treated organoids showed higher PI penetration than the control group with intact tight junctions, indicating compromised barrier integrity (Figures **5D, J**). Additionally, SYTOX staining also revealed reduced cell viability after diclofenac exposure (Figure **S9E**). These detrimental effects of diclofenac were markedly reduced when META was co-administered (Figures **5D, J,** and Figure **S9E**). Prompted by these findings, we then examined the expression of tight junction marker genes. META effectively prevented diclofenac-induced down-regulation of tight junction-associated transcripts, including Zo-1 and Ocln, compared to the diclofenac and Blank-MS groups (Figures **5K, L**). Because tight junction integrity is essential for preserving organoid structure, we next evaluated epithelial polarity. Under normal conditions, intact IOs typically exhibit basal-out polarity, with F-actin staining outlining the apical surface that faces the lumen (Figure **S9F**). In contrast, diclofenac treatment disrupted epithelial integrity and polarity, leading to structural disorganization and loss of the typical organoid morphology (Figures **5E, M**). Interestingly, META largely preserved epithelial polarity of IOs, whereas Blank-MS showed severe structural disruption similar to that seen with the diclofenac group (Figures **5E, M**). Collectively, these findings indicate that META effectively protects IOs against diverse cytotoxic agents, highlighting its therapeutic potential in preserving epithelial viability and barrier integrity under stress.

### Dual pH/ROS-response of META enhances drug delivery to the inflamed colon

**Figure 6:**
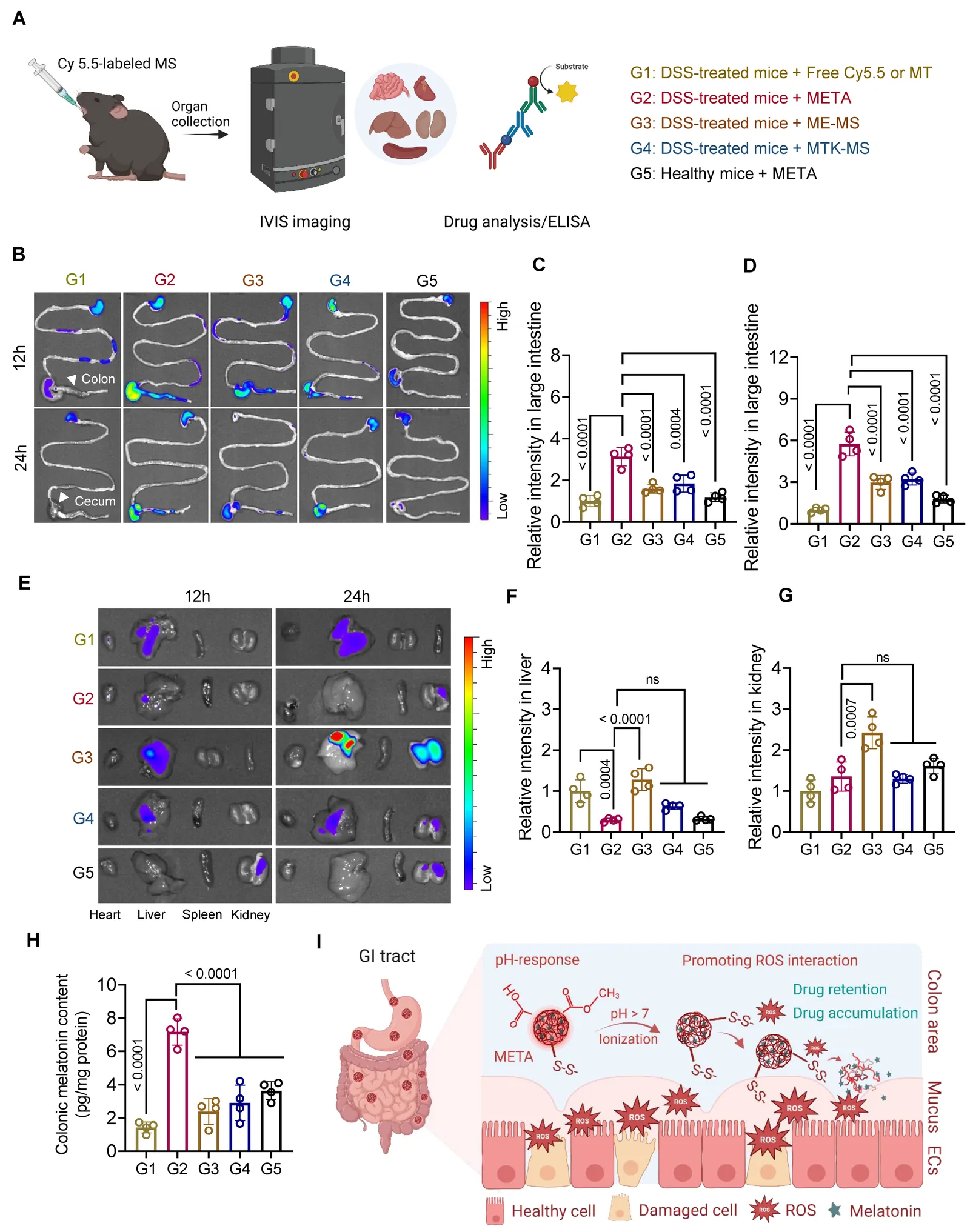
** Dual pH/ROS-response of META enhances drug delivery to the inflamed colon. A,** Illustration of biodistribution after oral treatment in mice using the *in vivo* imaging system (IVIS) and ELISA measurement. G1: DSS-treated mice + Free Cy5.5 or Free Melatonin (MT), G2: DSS-treated mice + META, G3: DSS-treated mice + ME-MS, G4: DSS-treated mice + MTK-MS, G5: Healthy mice + META. **B,** Fluorescence imaging shows the distribution of Cy5.5 in the GI tract. **C-D,** Relative fluorescence intensity in the large intestine 12 h (**C**) and 24 h (**D**) post-administration (n = 4 mice). **E,** Fluorescence distribution of Cy5.5 in major organs following different treatments. **F-G,** Relative fluorescence intensity 24 h post-administration in liver (**F**) and kidney (**G**) (n = 4 mice). **H,** Quantification of colonic melatonin content at 12 h post-administration by ELISA (n = 4 mice).** I,** pH- and ROS-interaction of META with the GI tract enhances drug retention and drug accumulation in the inflamed colon. Data in (**C, D, F, G, H**) are presented as mean ± s.d. Data in (**C, D, F, G, H**) are analyzed using one-way ANOVA with Tukey’s multiple comparisons test (significant p values are shown). **ns**, no significance. Figures **6A, I** were created with BioRender.com. MS: microsphere, MT: melatonin, ECs: epithelial cells.

Resistance to the harsh conditions of the GI tract and prolonged retention in the colon are critical attributes for orally administered microspheres used in colitis treatment. *In vitro* evidence indicated controlled melatonin release from META under GI tract-mimicking conditions in colitis, as previously demonstrated (Figure **3**). To further confirm the precise drug delivery of META, we investigated its fate following oral administration in healthy mice and DSS-induced colitis mice. Fluorescence images of Cy5.5-labeled microspheres were obtained using an *in vivo* imaging system (IVIS) at 12 and 24 h post-treatment (Figure **6A**). Free Cy5.5 and individual polymer-based microspheres were included for comparative purposes.

Orally administered free drug or microsphere exhibited distinct distribution patterns in the large intestine (Figure **6B**). In colitis mice, free Cy5.5 treatment showed low intensity at 12 h and was nearly undetectable at 24 h. Meanwhile, microsphere-based treatment revealed a stronger intensity in the colon area at both time points than that in the free Cy5.5 group. Notably, META demonstrated high efficiency of drug delivery to the inflamed colon with robust intensity of Cy5.5 compared to the individual polymer-based formulations (Figures **6B-D**).

These differences can be attributed to the interactive properties between each formulation and the altered GI environment in colitis. Free Cy5.5, lacking a protective carrier, is vulnerable to enzymatic degradation, premature systemic absorption in the small intestine, and poor mucosal adherence, which are all factors reducing its stability and retention in the GI tract 59, 60. Similarly, orally administered free melatonin faces comparable challenges, potentially limiting its therapeutic effectiveness. In contrast, META, a rationally designed delivery platform inspired by the physiological properties of the GI tract in colitis, could tune the interactions within this platform and effectively regulate drug release along with transit time in the colon. This design avoids either premature release due to high pH in the small intestine observed in ME-MS formulations or delayed release due to ROS-induced time-dependent response observed in MTK-MS formulations, both leading to diminished intensity due to rapid absorption or elimination from the colon before release. In healthy mice, META treatment exhibited a significantly lower colonic fluorescence intensity than that in the colitis mice (Figures **6B-D**). The lower ROS levels and intact mucosal barrier in healthy animals reduce META-mucus interactions, facilitating rapid clearance and significantly decreased Cy5.5 intensity (Figures **6B-D**). Thus, META enhanced drug retention and drug accumulation in the inflamed area, thereby potentially accelerating therapeutic response in colitis.

Major organs were also collected for fluorescence imaging to explore the fate of orally administered drugs. Notably, strong fluorescence signals were detected in the liver and kidney of the ME-MS group at both 12- and 24-h post-administration (Figures **6E-G**). This finding indicates that premature ME-MS release, triggered by the elevated pH in the small intestine, leads to systemic absorption and accumulation in off-target organs, resulting in off-target delivery and possible side effects. Similarly, free Cy5.5 showed substantial hepatic accumulation at both time points (Figures **6E-G**). As mentioned above, free Cy5.5 is unstable in the GI tract, undergoing rapid systemic absorption and degradation, which leads to poor intestinal retention and pronounced distribution to non-target tissues. In contrast, META and MTK-MS showed limited distribution in distant organs, attributable to controlled intestinal release profiles (Figures **6E-G**). However, delayed colonic release by the MTK-MS platform could reduce therapeutic efficacy due to elimination by peristaltic movement in the GI tract 61.

Melatonin in the colon was quantified in a mouse model administered oral free melatonin or microsphere formulations, following the same treatment protocol as that employed for Cy5.5 imaging, to confirm the targeted delivery capacity of META. The META group exhibited significantly higher colonic melatonin levels than both free melatonin and individual polymer-based microsphere groups in the colitis mice and also exceeded that observed in healthy mice treated with the same META platform (Figure **6H**). Collectively, these results demonstrate that the rationally designed META, featuring dual pH/ROS-responsiveness, enables precise and localized melatonin delivery to the inflamed colon, enhances drug retention, and promotes colonic drug accumulation via optimized interactions with GI tract physiology (Figure **6I**).

### META therapy effectively ameliorates UC through macrophage modulation

**Figure 7:**
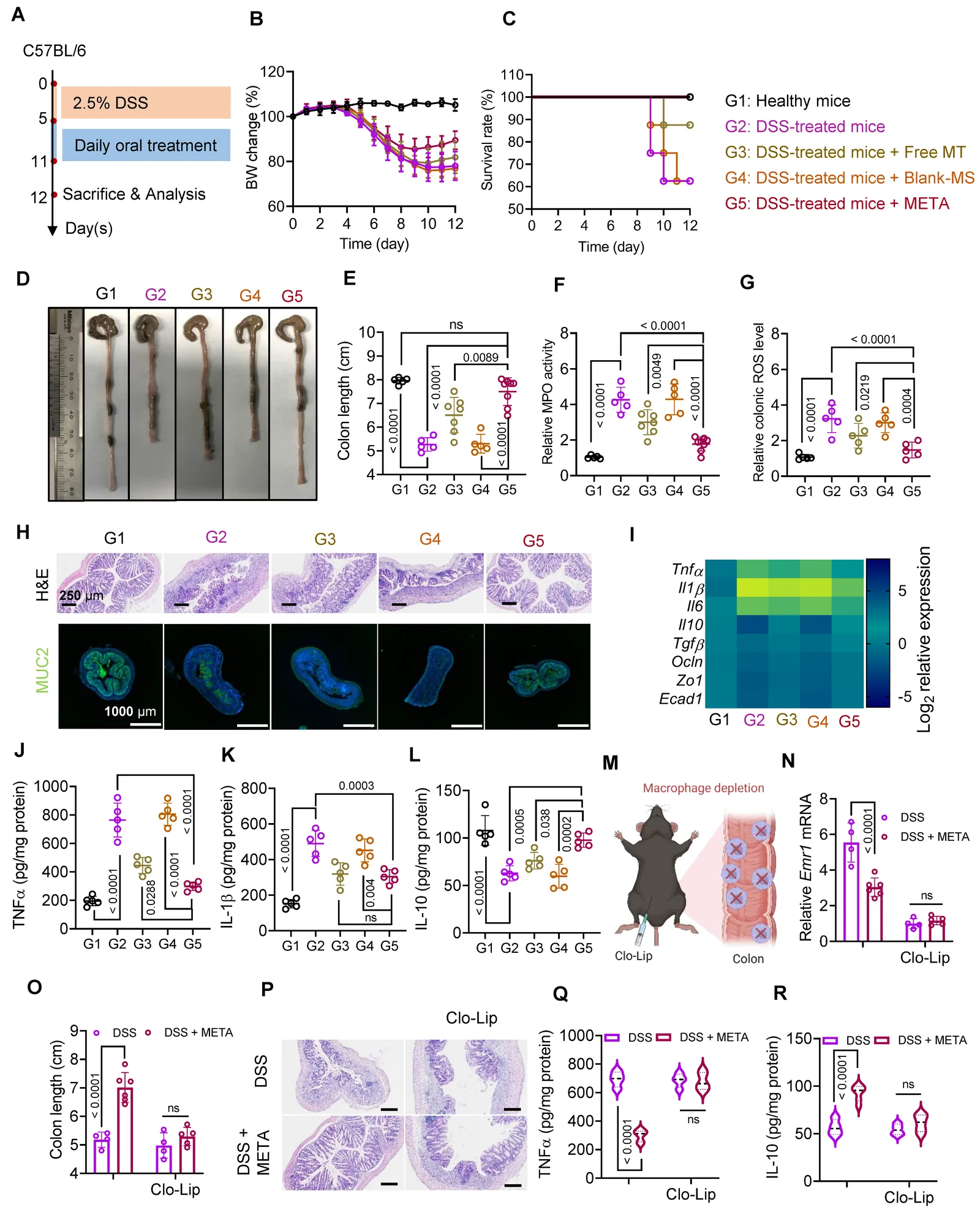
** META therapy effectively ameliorates UC through macrophage modulation. A,** Induction of the mouse colitis model through oral DSS delivery and subsequent drug treatment. G1: Healthy mice, G2: DSS-treated mice, G3: DSS-treated mice + free melatonin (MT), G4: DSS-treated mice + Blank-MS, G5: DSS-treated mice + META. **B,** Body weight change measurement (n = 5-8 mice). **C,** Survival rate of mice (n = 8 mice). **D,** Representative images of colon tissues. **E,** Colon length measurement (n = 5-8 mice). **F,** Myeloperoxidase (MPO) activity in colon tissues analysis (n = 5-8 mice). **G,** Colonic ROS measurement (n = 5-8 mice). **H,** Representative images of colon sections stained with H&E, scale bar: 250 µm (above panel) and representative immunofluorescence staining for MUC2 (green), scale bar: 1000 µm (below panel). **I,** Heat map of gene expression in colon tissues (n = 3-5 mice). **J-L**, ELISA measurement of colonic TNF-α (**J**), IL-1β (**K**), and IL-10 (**L**) (n = 5 mice). **M,** Macrophage depletion in mice via intraperitoneal injection of clodronate liposome (Clo-Lip). **N,** *Emr1* expression in colon tissue (n = 4-6 mice). **O,** Colon length measurement (n = 4-6 mice). **P,** Representative images of colon sections stained with H&E. Scale bar, 250 µm. **Q-R**, ELISA measurement of colonic TNF-α (**Q**) and IL-10 (**R**) (n = 4-6 mice). Data are presented as mean ± s.d. Data in (**E, F, G, J, K, L**) are analyzed using one-way ANOVA with Tukey’s multiple comparisons test (significant p values are shown). Data in (**N, O, Q, R**) are analyzed using two-way ANOVA with Tukey’s multiple comparisons test (significant p values are shown). Figure **7M** was created with BioRender.com. MS: microsphere, MT: melatonin.

The DSS-induced colitis mouse model was used to evaluate the therapeutic effects of META in UC (Figure **7A**). After 5 days of DSS treatment, mice then received daily doses of various treatments 19, 25, 27. As expected, the DSS-only and Blank-MS group exhibited the most severe decline in body weight, followed by the free melatonin groups. In contrast, mice treated with META lost less weight and began to recover earlier than the other colitis groups (Figure **7B**). Remarkably, META provided complete survival protection in DSS-treated mice, whereas other groups showed varying degrees of mortality. The free melatonin group had a survival rate of 87.5%, while the lowest survival rate of 62.5% was observed in colitis mice receiving no treatment or Blank-MS treatment (Figure **7C**).

Among all treatment groups, META-treated colitis mice exhibited the most pronounced recovery, with colon length restored to nearly that of healthy mice. Mice receiving free melatonin also had longer colons than untreated or Blank-MS-treated colitis mice, although the effect was less marked (Figures **7D, E**). In parallel, both myeloperoxidase (MPO) activity and ROS levels in the colon were significantly reduced in the META group compared with DSS group (Figures **7F, G**). H&E staining further showed that META provided the strongest protection, preserving crypt architecture and epithelial barrier integrity while reducing inflammatory cell infiltration more effectively than free melatonin, Blank-MS, or no treatment (Figure **7H, above panel**). Immunofluorescence staining also revealed higher mucin 2 (MUC2) expression in META-treated mice, indicating better maintenance of the intestinal barrier (Figure **7H, below panel**). Consistent with these findings, gene analysis showed that META inhibited M1 markers and increased M2 markers. META also promoted the expression of tight junction and adherens junction markers, supporting improved barrier integrity (Figure **7I** and Figure **S10**). These results were confirmed by ELISA, which showed that META reduced TNF-α and IL-1β levels while increasing IL-10 (Figures **7J-L**). To further assess macrophage polarization in colon tissue, we examined CD86 and CD206, which are surface markers associated with M1 and M2 macrophages, respectively. PCR results showed that META markedly suppressed CD86 expression and increased CD206 expression in the colons of colitis mice, further supporting its immunomodulatory effect (Figure **S11**).

We also examined changes in the gut microbiota after META oral administration (Figure **S12**). The results showed that DSS greatly altered the microbial profile compared with healthy controls. META partially restored a healthier microbial balance while reducing dysbiosis-related changes. These findings suggest that META may also work, at least in part, by modulating the gut microbiota, potentially through a melatonin-microbiota-intestinal barrier/macrophage axis.

Moreover, META therapy was safe *in vivo* and did not cause systemic toxicity after treatment (Figure **S13**). In addition, we evaluated the therapeutic stability of META to further support its practical applicability. The results showed that META effectively protected melatonin and controlled its release under SGF-mimicking conditions for at least 12 h (Figure** S14A**). After storage at room temperature for 3 months, META retained its spherical morphology, an average size of approximately 3 µm, a negative zeta potential, and melatonin loading capacity comparable to that of fresh META (Figure** S14B-D**). Furthermore, in a DSS-induced mouse colitis model, stored META showed therapeutic efficacy similar to fresh META, as demonstrated by diverse assessments (Figure** S14E-M).** Collectively, these findings highlight that META effectively ameliorated colonic inflammation in UC and exhibits strong potential for practical application.

### Macrophages as key mediators of META therapeutic activity in UC

To discover the functional role of tissue macrophages in META therapeutic efficacy, we depleted intestinal macrophages by intraperitoneal administration of clodronate liposomes (Clo-Lip) before and during the DSS-induced colitis model (Figure **7M**). Macrophage depletion was confirmed with the significant reduction in the mRNA of *Emr1*, the gene encoding F4/80, which is a key marker of macrophages, in the Clo-Lip-treated groups (Figure **7N**). Interestingly, in Clo-Lip-untreated groups, META treatment exhibited better recovery in colon length compared to DSS groups, whereas there was no significant difference between Clo-Lip-treated groups, and both resembled the Clo-Lip-untreated DSS group (Figure **7O**). Histological assessments showed the severe condition of colon tissue with damaged crypt structure and epithelial barriers in Clo-Lip-treated groups, including DSS and META groups (Figure **7P**). Furthermore, clodronate liposome treatment reversed META’s suppressive effect on the inflammatory cytokine TNF-α compared to the macrophage-intact META group (Figure **7Q**). Macrophage depletion also abolished IL-10 production in both META and DSS groups (Figure **7R**). Collectively, these findings indicate that the therapeutic effects of META are largely dependent on the presence of colonic macrophages.

### Dual pH/ROS-response in META is critical for outstanding therapeutics against UC

The individual polymer-based microspheres, including ME-MS and MTK-MS, also showed potential therapeutic effects under *in vitro* conditions (Figure **S15** and Figure **S16**). This is consistent with the fact that, *in vitro*, melatonin can be readily released and directly interacts with cells in a controlled environment. However, *in vivo* delivery is much more challenging. After oral administration, microspheres must pass through several barriers in the GI tract before reaching the inflamed colon. The acidic gastric environment and the risk of premature release in the small intestine may limit the ability of single-polymer systems to achieve efficient colonic delivery and sustained therapeutic effects. As shown above, the dual pH/ROS-responsive META platform improved drug delivery to the inflamed colon more effectively than the single platforms. We next evaluated whether META could also provide better therapeutic outcomes in the DSS-induced colitis model than the individual polymer-based microspheres, ME-MS and MTK-MS (Figure **S17A**). All treatment groups improved body weight compared with untreated DSS mice, but META induced earlier and more pronounced recovery (Figure **S17B)**. Survival rates were also improved in all treatment groups, with META and ME-MS completely preventing mortality in colitis mice (Figure **S17C**). In addition, META restored colon length more effectively than DSS alone, while ME-MS and MTK-MS showed intermediate improvement (Figures **S17D, E**). MPO activity further indicated that META suppressed neutrophil infiltration and inflammation more effectively than either ME-MS or MTK-MS (Figure **S17F**). At the molecular level, META produced the strongest therapeutic response, with clear downregulation of pro-inflammatory markers (*Tnfα* and* Il1β*), upregulation of anti-inflammatory (*Il10*) and tight junction genes (*Ocln* and *Zo1*) compared to single polymer-based formulations (Figure **S17G**). Overall, these findings show that the rational dual pH/ROS-responsive design of META enables more precise melatonin delivery in the GI tract and leads to better therapeutic efficacy than the individual polymer-based systems.

## Discussion

Among the existing treatments for UC, the highest patient compliance is observed with oral therapies, including 5-ASA, corticosteroids, immunosuppressants, and biologics. However, these often encounter limited colon-specific targeting, systemic side effects, and suboptimal efficacy due to the complex pathophysiology of UC 2, 29, 30. These treatments primarily address downstream symptoms or isolated pathways, frequently failing to interrupt the disease’s vicious cycle or to restore intestinal barrier integrity comprehensively 10, 62.

Melatonin is an endogenous hormone produced in the GI tract and is an attractive therapeutic candidate because of its intrinsic multiple beneficial effects, including anti-inflammatory activity, antioxidant effects, and protection of the intestinal barrier, as shown in this study. Unlike many small-molecule drugs that do not directly match the underlying mechanisms of UC, melatonin acts on key upstream drivers such as immune imbalance and oxidative stress. Because it is an endogenous molecule, melatonin may be highly favorable to the GI tract condition, supporting tissue repair and recovery. However, the therapeutic potential of oral melatonin is limited by its early absorption and poor accumulation at inflamed colonic sites, which reduces its effectiveness and may also increase systemic side effects 27, 29, 31, 32.

In this study, we indicated that melatonin plays an important role in colonic inflammation. We also addressed the main limitation of oral melatonin delivery by using the META platform. This allows melatonin to be delivered more precisely, helping regulate immune responses and protect the intestinal barrier. However, to better assess the targeting performance of META, future studies will need more advanced methods such as LC-MS to more accurately evaluate the pharmacokinetics of the drug and microspheres.

Mechanistically, META could modulate macrophage polarization and reinforce the intestinal epithelial barrier. Deletion of intestinal macrophages reduces META’s therapeutic benefit, underscoring the pivotal role of macrophage-melatonin interactions in disease amelioration. Further studies should clarify the interaction between melatonin, an endogenous agent, and macrophages, an important immune cell type implicated in UC. Deep mechanistic insights into these interactions could inform drug selection and the design of delivery systems such as META, which promote native therapeutic effects at the site of inflammation. This integrated approach may yield superior therapies combining precision delivery with biological rationality, minimizing off-target effects, and maximizing efficacy.

In addition to the mechanisms assessed in this study, melatonin has been reported to regulate gut microbiota composition in experimental colitis models, and this action is increasingly recognized as critical to its therapeutic effect 23, 63. Melatonin-mediated improvements in barrier function, mucin production, and antimicrobial peptide induction contribute to shaping a healthy microbial environment, while shifts in microbiota further modulate host immune responses 64. Our PCR-based microbiota abundance analysis showed that DSS caused a pronounced dysbiotic shift, whereas META partially reversed these changes and moved the microbial profile toward that of healthy mice. In particular, the recovery of beneficial taxa together with the attenuation of DSS-associated microbial alterations supports a potential role for the gut microbiota in the therapeutic action of META. However, these analyses were based on limited information regarding the gut microbiota and therefore cannot fully elucidate the underlying mechanisms of META. In future studies, more comprehensive 16S rRNA sequencing analyses, including microbiota composition, α/β diversity, and key taxonomic shifts, will be of great interest for characterizing the “melatonin-microbiota-intestinal barrier/macrophage” axis, and may reveal further synergistic mechanisms underlying META therapeutic benefit in UC.

A notable advancement of this study is the use of IOs as an advanced *in vitro* model to evaluate META’s protective effects on the intestinal barrier under inflammatory and oxidative stress conditions. IOs could closely recapitulate the complex epithelial architecture, cell heterogeneity, and physiological responses of the intestine 65. Our META significantly preserved organoid integrity from different damage factors. This organoid-based assessment strongly supports the translational potential of META from *in vitro* experiments to *in vivo* outcomes.

The DSS-induced colitis model reflects many key features of human UC, which supports the relevance of our findings. However, mouse models still have clear limitations, because immune responses and disease progression can vary between species. This is especially important for META, since it is designed to respond to the altered GI environment during colitis. Before clinical translation, future studies in larger animal models and non-human primates will be needed to confirm its efficacy and safety.

Other than its biological success, the META drug could also be produced using an easy and scalable emulsification technique, which would increase its translational capability. Through the advancement of technologies such as microfluidic technology, the META system could be improved to increase its capacity to load drugs and its encapsulation effectiveness, thus making large-scale production cost-effective. The easy production technique will solve one of the key problems facing the translational capability of pharmaceuticals 66, bridging the gap from the lab to the bedside. Additionally, since the META drug delivery system is modular, it could be modified to deliver a wide variety of therapeutic drugs other than melatonin for other conditions. Besides, META is generated from biocompatible and safe materials with solid evidence. Together, these features give META both useful functionality and good translational potential.

In conclusion, this study provides mechanistic insight with practical designs to develop an orally effective delivery system. META improves melatonin delivery, which regulates macrophage activity and the intestinal barrier in the inflamed colon. Its simple preparation and consistent performance suggest that it could be a promising approach for UC and other inflammatory GI diseases.

## Materials and Methods

### Animals and cell culture

Male C57BL/6 mice (8–10-week-old) were purchased from Orient Bio (Republic of Korea). All animal experiments were performed following the national ethical guidelines and were approved by the Institutional Animal Care and Use Committee at Sungkyunkwan University (Suwon-si, Gyeonggi-do, Republic of Korea) (Approval no. SKKU IACUC 2023-09-28-1).

The RAW 264.7 murine macrophage cell line (ATCC) and the human intestinal epithelial cell line Caco-2 (ATCC) were cultured in Dulbecco’s Modified Eagle’s Medium (DMEM; ByLABS, cat# BY0011) supplemented with 10% fetal bovine serum (FBS; Gibco, cat# 12483020) and 1% penicillin-streptomycin solution (ByLABS, cat# BY1411), at 37 °C and 5% CO_2_.

### Fabrication and characterization of melatonin-loaded microspheres

Melatonin-loaded microspheres were synthesized using an emulsification solvent-evaporation technique. Briefly, 90 mg of polymer, either thioketal polymer, Eudragit^®^ FS 30 D (Evonik, Germany, CAS# 26936-24-3 and Lot# C220165001), or an equal mixture of both, was dissolved with 10 mg of melatonin (Thermo Fisher Scientific, cat# J62452.14) in 1 mL of dichloromethane (DCM). This organic phase was slowly added dropwise to 5 mL of an aqueous 1% (w/v) polyvinyl alcohol (PVA) solution under homogenization at 13,000 rpm for 5 min to generate an oil-in-water emulsion. The emulsion was then transferred to 60 mL of 1% PVA and stirred at RT for 5 h to allow solvent evaporation. The resulting microspheres were then recovered by centrifugation, washed, and freeze-dried. Their morphology was examined by scanning electron microscopy (SEM; JEOL, JSM-IT800), particle size distribution was determined using laser diffraction (Beckman Coulter LS 13320, CA, USA), and zeta potential was measured with a Zetasizer (Malvern, UK). Additionally, we prepared a blank microsphere (Blank-MS), used as a control of META, containing Eudragit^®^ FS 30 D and thioketal polymer without melatonin, for further functional assessments.

The amount of melatonin was analyzed by high-performance liquid chromatography (HPLC; Waters, USA) using a C18 column (5 µm, 150 × 4.6 mm; GL Science). Mobile phases for HPLC were prepared using acetonitrile and water (ratio 70:30, v/v) with a flow rate of 1 mL/min. Melatonin detection was done using a photodiode array detector at a wavelength of 220 nm. The loading capacity and entrapment efficiency were calculated using the formulas given in Table **S2**.

To assess the pH/ROS-responsive degradation of META, microspheres were sequentially incubated in simulated gastric fluid (SGF; pH 2.5) for 2 h, simulated intestinal fluid (SIF; PBS pH 7.2) for 4 h, and simulated colonic fluid (SCF; PBS pH 7.4) containing 1 mM H_2_O_2_ to mimic elevated ROS. Changes in microsphere surface morphology were examined by SEM. Melatonin release under these simulated GI conditions was quantified by HPLC. The release profiles of different formulations were compared using the similarity factor *f_2_* (Table **S2)** with *f_2_* values below 50 indicating dissimilar dissolution profiles 67.

### *In vitro* ROS assay

RAW 264.7 macrophage and Caco-2 epithelial cells were seeded in 96-well plates at a density of 1 × 10^4^ cells per well. RAW 264.7 cells were activated with lipopolysaccharide (LPS, 500 ng/mL, cat# 2880-25MG), while Caco-2 cells were exposed to 100 µM H_2_O_2_. After 12 h of stimulation, cells were incubated with either free melatonin, Blank-MS, or the META for 24 h. Subsequently, the cells were stained with DCFH-DA (Sigma-Aldrich, cat# 35845-1G) for 15 min, followed by three washes with PBS. Fluorescence imaging was performed using a fluorescent microscope (Nikon, Japan), while quantitative analysis was conducted with a microplate reader (TECAN, Austria) at an excitation wavelength of 485 nm and emission wavelengths of 535 nm 68, 69.

### *In vitro* anti-inflammatory assay

A co-culture model was established for Caco-2 and RAW 264.7 macrophages using 6-transwell insert plates (0.4 µm pore size, SPL), following a previously described method 70. RAW 264.7 macrophages were then activated with LPS for 12 h. Subsequently, Blank-MS or META were treated to the basolateral chamber and incubated for 24 h. Supernatants were withdrawn for cytokine analysis by ELISA, including TNF-α (BioLegend, cat# 430904), IL-1β (BioLegend, cat# 432601), IL-6 (BioLegend, cat# 431304), and IL-10 (BioLegend, cat# 431414). Cells were harvested to evaluate inflammatory gene expression using RT-PCR.

### *In vitro* permeability assessment

For *in vitro* cellular permeability studies, Caco-2 cells (1 × 10^5^ cells) were seeded in the apical chamber with a pore size of 0.4 µm of 24-transwell plates (Corning, USA). Upon reaching confluence and forming a monolayer, cells were treated with 100 µM H_2_O_2_ for 24 h. The monolayer cells were then washed twice with PBS and incubated with either Blank-MS or META for an additional 24 h. After treatment, monolayer cells were washed twice with PBS, and 200 µL of fluorescein isothiocyanate-dextran 10 kDa (FD10, Sigma-Aldrich, cat# FD10S-100MG) at 1 mg/mL in HBSS was added to the apical chamber. Samples from the basolateral chamber were collected after 2 h, and FD10 fluorescence was determined using a microplate reader (TECAN, Austria) at excitation/emission wavelengths of 485/525 nm 71, 72. Additionally, TEER (Table **S2**) was measured at predefined time points using an EVOM2 epithelial voltohmmeter (World Precision Instruments) to monitor monolayer integrity throughout the assay 73.

### Culturing IOs and image-based analysis

IOs were derived from mouse small intestine and cultured following established protocols 74*.* Only organoids from passage 4 exhibiting stable growth and typical morphology were selected for experiments. Organoids were treated with varying concentrations (5-40 µg/mL) of Blank-MS or META, as well as with inflammatory stimuli including interferon-γ (IFN-γ; 10 ng/mL, Peprotech, Rockyhill, NJ), tumor necrosis factor-α (TNF-α; 10 ng/mL, Peprotech), interleukin-1β (IL-1β; 10 ng/mL, Peprotech), diclofenac (100 or 300 µM, MedChem Express, Monmouth Junction, NJ), and tert-butyl hydroperoxide (tBHP; 200 µM, Sigma-Aldrich, St. Louis, MO). Organoid morphology and viability were assessed by imaging with a BioTek Cytation 5 Cell Imaging Multi-Mode Reader (Agilent, Santa Clara, CA), and images were processed using Gen5 software with the image analysis module.

### Assessments of IO viability and permeability

For growth assays, 50 organoids per well were cultured into 96-well plates for 3 days. Bright-field images were captured at the time of seeding (day 0) and on day 3. Relative growth was quantified by calculating the ratio of the organoid-covered area on day 3 to that on day 0. SYTOX Green (Thermo Fisher Scientific, Waltham, MA) staining was performed to assess organoid viability. A final concentration of 500 nM SYTOX Green was added to the culture medium and incubated for 15 min. Fluorescent images were then acquired to quantify the proportion of dead organoids, defined by SYTOX Green-positive staining. To evaluate cell-cell junctional integrity following damage, a permeability assay using propidium iodide (PI) was conducted 75. IOs were cultured in 96-well plates (50 organoids per well) for 48 h and then treated with diclofenac, with or without META. After 24 h, PI (5 µg/mL; Thermo Fisher Scientific) was added to each well and incubated for 20 min at 37 °C. Bright-field and red fluorescence images were sequentially captured to visualize total organoid structures and PI-positive (permeable) regions, respectively.

### Oxidative stress induction in IOs and ROS assessments

For real-time viability assessment, 5 µM CellROX Orange Reagent (Thermo Fisher Scientific) was added to organoid cultures on day 3 and incubated at 37 °C for 20 min. Following this, organoids were treated with t-BHP in the presence of either Blank-MS or META. Organoid images were acquired at 10-min intervals for a total duration of 90 min and analyzed to calculate the intensity of CellROX-positive organoid area over time. Meanwhile, the ROS levels in the IOs were assessed using flow cytometry. After 1-h exposure to tBHP in the presence of microspheres, organoids were incubated with 5 µM CellROX Deep Red Reagent (Thermo Fisher Scientific) for 20 min at 37 °C. Organoids were then harvested, dissociated into single cells via trypsinization, and subjected to flow cytometric analysis using an Accuri C6 Plus flow cytometer (BD Biosciences, Ashland, OR, USA).

### DSS-induced acute colitis model and treatment

Acute UC was induced in male C57BL/6 mice (8–10-week-old) by administering 2.5% (w/v) DSS (36-50 kDa, MP Biomedicals, cat# 160110) for 5 days 54, 76. Following DSS treatment, mice received daily oral gavage of either free melatonin (5 mg/kg/day) 25, melatonin-loaded microspheres (equivalent to 5 mg/kg/day of free melatonin), or Blank-MS for 7 days. Body weight and clinical symptoms were recorded daily until mice were sacrificed. The disease activity index (DAI) was measured following Table **S3**. On day 12, mice were euthanized, and colon tissues were collected for cytokine quantification, gene expression analysis, and histological assessments. In addition, colonic contents were harvested, and total bacterial DNA was extracted using a QIAamp FAST DNA Stool Mini Kit (Qiagen, Germany, Cat #51604) for qRT-PCR analysis to evaluate the abundance of disease-associated bacterial candidates (Table **S4**).

### *In vivo* intestinal permeability assay and MPO activity assay

Gut barrier function was assessed in C57BL/6 mice using the FD10 permeability assay. Acute colitis was induced, and treatments were administered as described previously. Mice were fasted for 6 h prior to the assay and then orally gavaged with 150 µL of 80 mg/mL FD10 solution. Four hours post-gavage, blood samples were collected, and serum FD10 levels were quantified by fluorescence measurement at excitation/emission wavelengths of 485/525 nm 54, 71, 77. The myeloperoxidase (MPO) activity was evaluated using a previously described method 43, 74.

### Colonic ROS measurement

Colon tissues were rinsed twice with PBS (pH 7.4) and cut into small fragments. The tissue pieces were then digested in serum-free RPMI-1640 medium containing 0.1% collagenase P (Roche, cat# 11213857001) and 0.01% DNase I (Roche, cat# 11284932001) at 37 °C for 30-45 min with gentle shaking. The resulting suspension was passed through a 40-µm strainer to obtain single-cell suspensions. The cells were then washed, resuspended in culture medium, and incubated with 20 µM DCFH-DA at 37 °C for 15 min in the dark. After removing the excess dye by washing, intracellular ROS levels were quantified by a microplate reader at excitation and emission wavelengths of 485 and 535 nm, respectively. Fluorescence intensity was normalized against cell number.

### Quantitative real-time polymerase chain reaction

Total RNA was extracted from Caco-2 cells, RAW 264.7 cells, or colon tissues using TRIzol reagent (Invitrogen, cat# 15596018). RNA concentration and purity were quantified using a NanoDrop 2000 spectrophotometer (Thermo Fisher Scientific). cDNA was synthesized using the RevertAid First Strand cDNA Synthesis Kit (Thermo Fisher Scientific, cat# K1622). qRT-PCR was then performed on a QuantStudio real-time PCR system using SYBR Green Master Mix (Applied Biosystems, cat# 4309155). Relative gene expression was calculated using the ΔΔCt method, with *Gapdh* or *Actb* serving as internal reference genes. Primer sequences were obtained from Bioneer Corporation (Daejeon, Republic of Korea) and are listed in Table **S5**.

### Enzyme-linked immunosorbent assay

Cytokine and melatonin collected from cells and colon tissues were quantified using ELISA kits (for melatonin, mouse ELISA kit, ELK Biotechnology, cat# ELK9480) following the instructions from the manufacturer. Cells or tissues were lysed in RIPA buffer containing 1% protease inhibitor and 1% EDTA (Thermo Fisher Scientific). Absorbance was then read at 450 nm.

### *In vivo* imaging analysis

To evaluate the targeted delivery capability of the META platform, Cy5.5-labeled microspheres and free Cy5.5 were orally administered to healthy and DSS-induced colitis mice at equivalent Cy5.5 doses. For labeling, Cy5.5 dye was incorporated into the microsphere formulation during the preparation process under the same conditions used for the corresponding blank or drug-loaded microspheres. After preparation, the Cy5.5-labeled microspheres were collected and washed to remove unbound dye. The Cy5.5 content in each microsphere formulation was quantified by fluorescence measurement to ensure consistent labeling and to allow dose adjustment for equivalent Cy5.5 administration across all groups. Mice were sacrificed at 12 and 24 h post-administration. The GI tract and major organs were harvested for fluorescence imaging using an In Vivo Imaging System (IVIS, Caliper Life Sciences) equipped with a Cy5.5 filter.

### Depletion of intestinal macrophages

In brief, 200 µL of clodronate-liposome (Clo-Lip) (Encapsula NanoSciences LLC, Brentwood, USA) was intraperitoneally administered to mice 4 days before initiating DSS-induced colitis to deplete resident macrophages, and subsequently every 3 days throughout the protocol to eliminate infiltrating macrophages 78, 79.

### AI tool

Perplexity was used to improve the language and readability of the **Materials and methods** section. Specifically, we first drafted this section ourselves and then used Perplexity to refine it for the above purposes. After using this tool/service, the authors carefully reviewed and edited the content as needed and take full responsibility for the content of the published article.

### Statistical analysis

GraphPad Prism was used for statistical analyses. Unpaired two-tailed t-tests assessed differences between two groups, while comparisons involving three or more groups were conducted using one-way ANOVA or two-way ANOVA followed by post hoc tests (Tukey’s multiple comparison or false discovery rate, FDR correction). FDR-controlled tests report q values, whereas other tests present p values. Statistical significance was considered as p or q < 0.05.

## Supplementary Material

Supplementary methods, figures and tables.

Supplementary figure 9, movie.

## Data Availability

The data supporting the findings of this study are available from the corresponding author upon reasonable request.

## Abbreviations

AJ: adherens junction
Clo-Lip: clodronate liposome
DSS: dextran sulfate sodium
EC: epithelial cell
ELISA: enzyme-linked immunosorbent assay
FD10: FITC-Dextran 10 kDa
GI: gastrointestinal
H&E: hematoxylin and eosin
IO: intestinal organoid
IVIS: *in vivo* imaging system
LPS: lipopolysaccharide
ME-MS: Melatonin-Eudragit microsphere
META: Melatonin-Eudragit-Thioketal polymer-based Assembly
MTK-MS: Melatonin-Thioketal microsphere
MT: melatonin
PI: propidium iodide
ROS: reactive oxygen species
SCF: simulated colonic fluid
SEM: scanning electron microscopy
SGF: simulated gastric fluid
SIF: simulated intestinal fluid
TEER: transepithelial electrical resistance
TJ: tight junction
UC: ulcerative colitis
