# Supplementary material for: Colon-targeted oral delivery of melatonin for regulating macrophage activity and intestinal barrier in ulcerative colitis
Source: Theranostics. 2026 Aug 24;16(15):8860–79. doi: 10.7150/thno.133456 (PMC13549380; doi:10.7150/thno.133456)
Supplement: Supplementary file 1 [file thnov16p8860s1.pdf]

Supporting information for

Original article

Colon-targeted oral delivery of melatonin for regulating macrophage activity and intestinal barrier in ulcerative colitis

Supplementary methods

siRNA transfection on RAW 264.7 macrophages

RAW 264.7 cells (5×10^5 cells/well) were seeded in 6-well plates in 2 mL DMEM overnight. For transfection, 3 µL of 20 µM STAT3 siRNA (cat# 20848-3) and 3 µL lipofectamine 2000 (Invitrogen, cat# 11668-019) were separately diluted in 100 µl Opti-MEM (Gibco, cat# 31985-070), incubated for 5 min at RT, combined, and further incubated for 20 min to form complexes. Cell culture medium was replaced with 1.8 mL fresh DMEM, and 200 µL transfection complexes were added dropwise. After a transfection period of 48 h, cells were subjected to downstream assays such as LPS stimulation. Knockdown efficiency was verified by qRT-PCR and Western blot (> 70% STAT3 reduction). Scrambled siRNA (SN-1001) served as a negative control [1, 2].

Western blot assay

Briefly, RAW 264.7 cells were lysed in RIPA buffer containing 1% protease inhibitor and 1% EDTA (Thermo Fisher Scientific). The lysates were homogenized and centrifuged to collect protein samples, which were then determined and subjected to SDS-PAGE and membrane transfer. The membrane was then blocked with BSA 5% and sequentially incubated with primary antibodies against STAT3 (CST, cat# 4904S), p-STAT3 (CST, cat# 9145S), and GAPDH (ABclonal, cat# A19056), followed by HRP-conjugated secondary antibodies (Invitrogen, cat# 31460). Protein expression was visualized using enhanced chemiluminescence (ECL) detection.

In vitro cytotoxic assay

Caco-2 epithelial cells and RAW 264.7 macrophages were maintained in complete medium. Cells (1×10^4 per well) were seeded in 96-well plates overnight. Subsequently, cells were treated with serial concentrations of free melatonin or Blank-MS for 24 h. Cell viability was then assessed using the WST-8 assay (Biomax, cat# QM2500) following the instructions from the manufacturer.

Synthesis and confirmation of thioketal polymer

The thioketal polymer with ROS-responsiveness was synthesized via step-growth polymerization, as described in our previous study [3]. Briefly, 245 mg 4,4′-bis(mercaptomethyl)biphenyl (Sigma-Aldrich, cat# 716049) was dissolved in 15 mL toluene containing 250 μL 2,2-dimethoxypropane (DMP; Sigma-Aldrich, cat# D136808). The solution was heated to 75 °C, after which p-toluenesulfonic acid catalyst (2 mg in 250 μL ethyl acetate; Sigma-Aldrich, cat# 402885) was added. Additional DMP in toluene (500 μL aliquots, prepared as 500 μL DMP in 10 mL toluene) was provided every 30 min over 10 h, followed by a final 500 μL addition of DMP. The reaction was then stirred overnight. Then, cold hexane (−20 °C) was used to precipitate the synthesized polymer. The product was then washed twice with distilled water and dried to obtain a brown viscous product. Polymer structure was analyzed and confirmed by ^{1}H and ^{13}C NMR spectroscopy (500 MHz, Varian Unity Inova).

Measurement of luminal pH of the gastrointestinal tract in mice

Healthy mice and DSS-induced colitis mice were fasted overnight and euthanized by CO_2 inhalation. The gastrointestinal tract was rapidly dissected, and luminal contents were collected from distinct regions (stomach, small intestine, large intestine). For samples with insufficient volume, 50 μL sterile water was added, gently homogenized, and mixed. Luminal pH was measured immediately using a calibrated micro pH electrode to limit post-mortem alterations. Region-specific pH gradients were recorded directly from GI contents [4].

***In vivo* safety of META**

Healthy mice received daily oral administration of META for 7 consecutive days. Serum was collected for biochemical analysis of liver function (GOT, GPT; Asan Set Assay kit) and kidney

function (BUN; Colorimetric Detection Kit, Invitrogen). Major organs were harvested for histological evaluation.

Supplementary figures

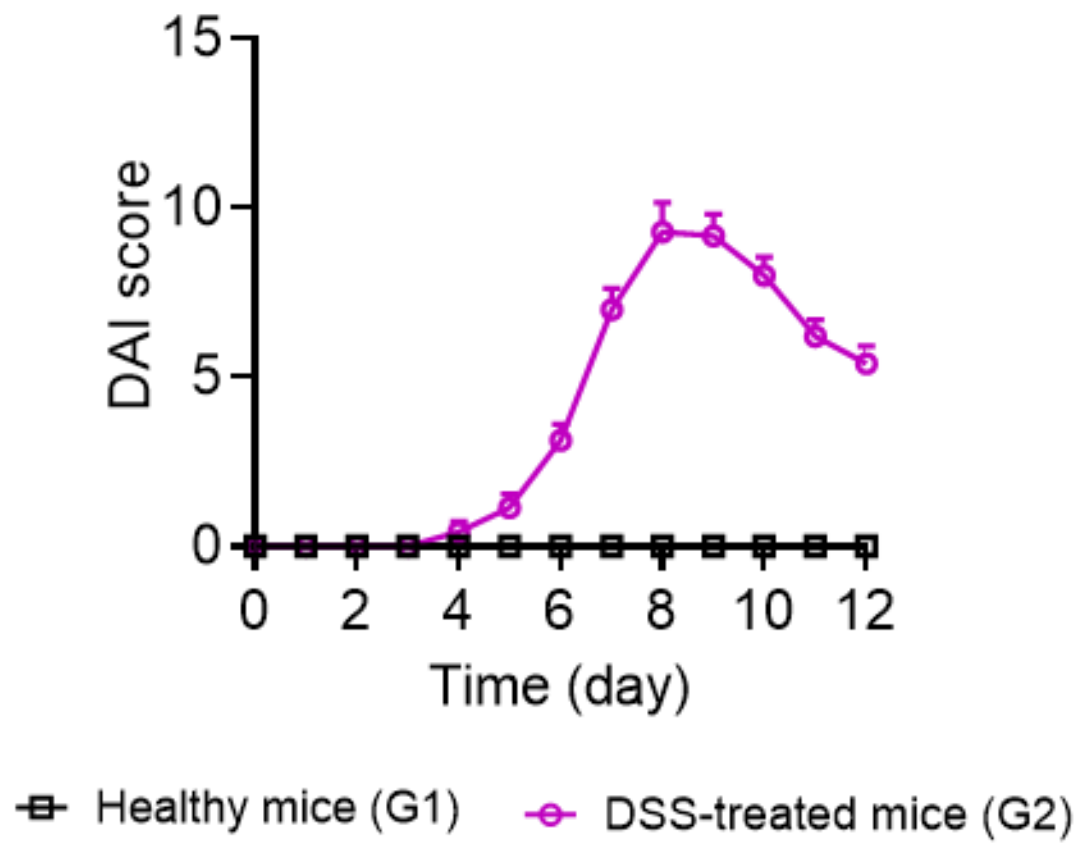

Figure S1. DAI score of DSS-induced mice colitis (n = 4-7 mice). Data are presented as mean ± s.e.m.

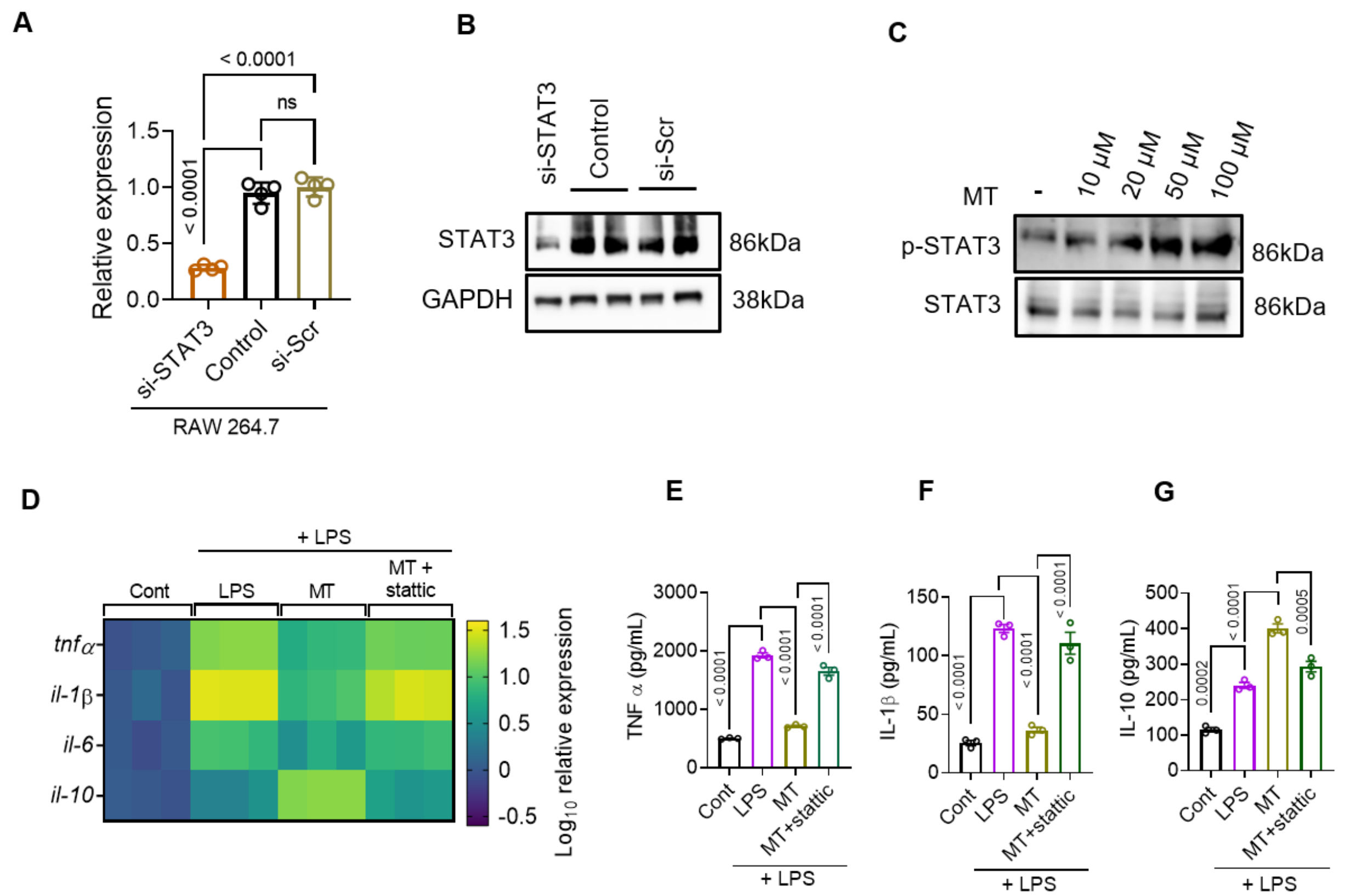

Figure S2. Melatonin regulates macrophage activity through STAT3 signaling. A, Expression of STAT3 mRNA in RAW 264.7 cells (n = 4). **B,** Expression of STAT3 protein in RAW 264.7 cells. **C,** Melatonin activates STAT3 in LPS-treated RAW 264.7 cells. **D,** Modulatory effects of melatonin on macrophage polarization after STAT3 inhibition, using stattic 5 μM, quantified by qRT-PCR (n = 3). **E-G,** ELISA quantification of extracellular cytokines, including TNF-α, IL-1β, and IL-10, respectively (n = 3). Data in (**A, E, F, G**) are presented as mean ± s.d. Data are analyzed using one-way ANOVA with Tukey's multiple comparisons test.

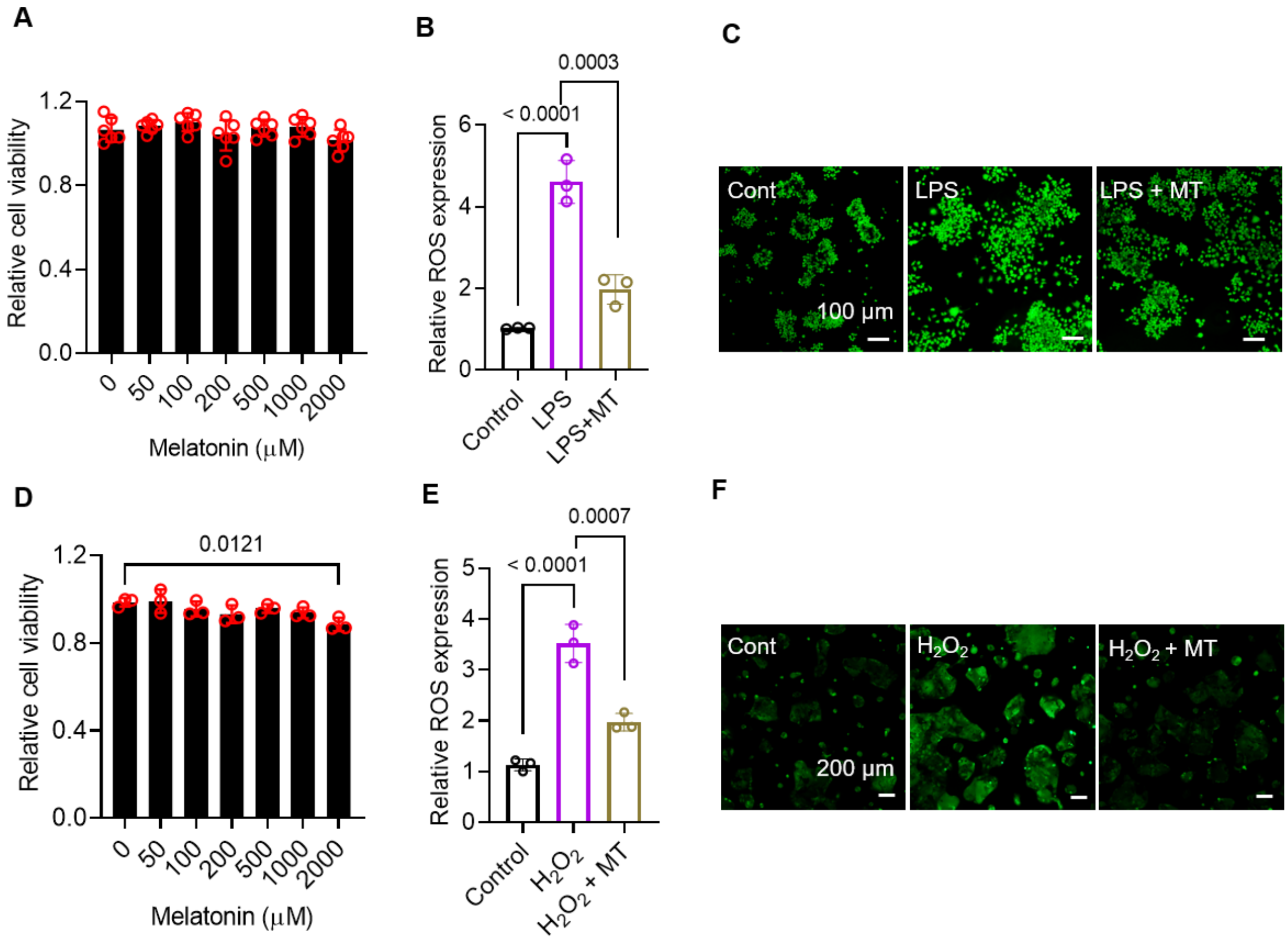

Figure S3. *In vitro* cytotoxicity of melatonin and its antioxidant effects. A, Cytotoxicity of melatonin on RAW 264.7 macrophages (n = 6). **B,** The antioxidative capacity of melatonin in RAW 264.7 cells (n = 3). **C,** Imaging ROS levels in RAW 264.7 cells. Scale bar 100 μm. **D,** Cytotoxicity of melatonin on Caco-2 epithelial cells (n = 3). **E,** The antioxidative capacity of melatonin in Caco-2 cells (n = 3). **F,** Imaging ROS levels in Caco-2 cells. Scale bar 100 μm. Data in (**A, B, D, E**) are presented as mean ± s.d. Data in (**A, D**) are analyzed using an unpaired two-tailed t-test (significant p values are shown). Data in (**B, E**) are analyzed using one-way ANOVA with Tukey's multiple comparisons test (significant p values are shown).

A

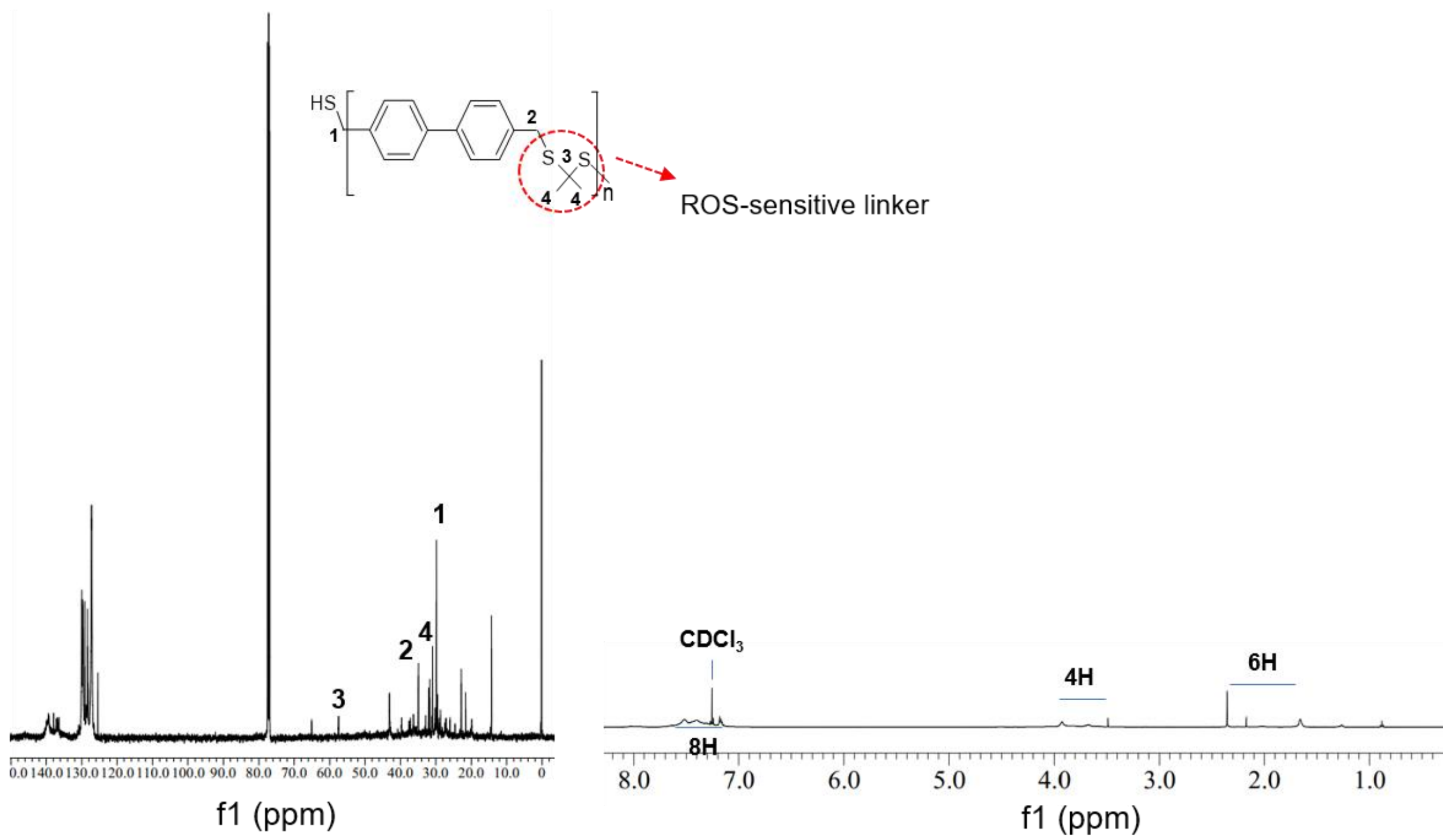

B

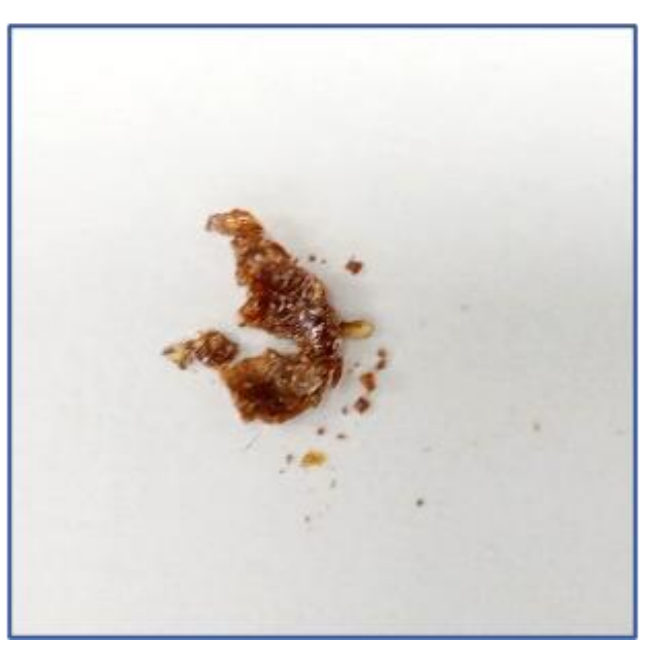

Figure S4. Synthesis and confirmation of thioketal polymer. A, Chemical structure of thioketal polymer was confirmed by ^{1}H and ^{13}C NMR spectroscopy. **B,** Image of synthesized thioketal polymer. Chemical formulation was created with ChemSketch software (ACD/Labs).

A

Formulation	Melatonin (%)	FS 30 D (%)	Thioketal polymer (%)
F1 (ME-MS)	10	90	0
F2 (META)	10	45	45
F3 (MTK-MS)	10	0	90

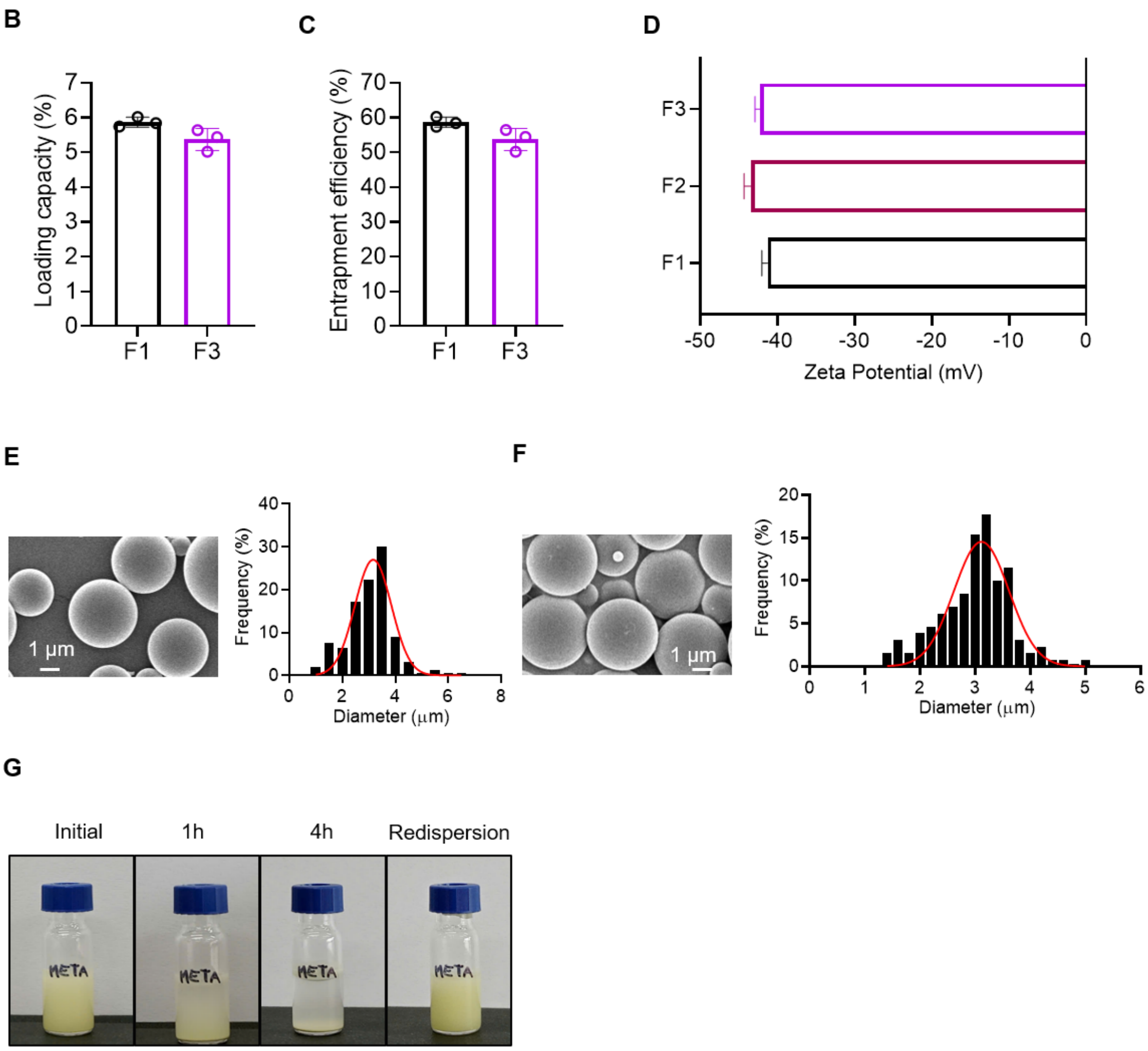

Figure S5. Optimization of microsphere formulations. A, Different formulations of microspheres for optimization. **B,** Loading capacity of ME-MS and MTK-MS (n = 3). **C,** Entrapment efficiency of ME-MS and MTK-MS (n = 3). **D,** Zeta potential of microspheres (n =

3). **E,** Morphology and size distribution of ME-MS. **F,** Morphology and size distribution of MTK-MS. Scale bar: 1 μm. **G,** META dispersibility in water.

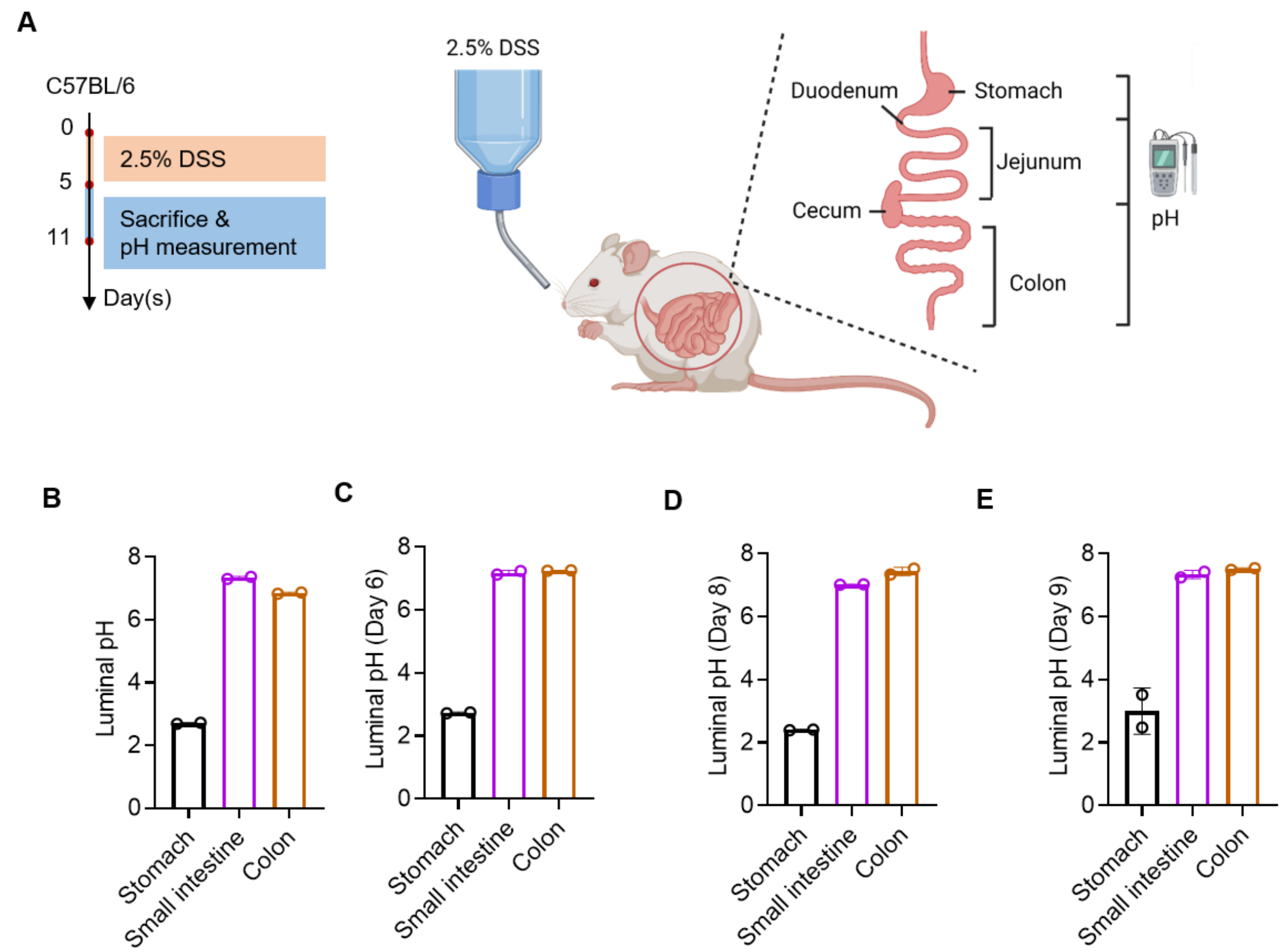

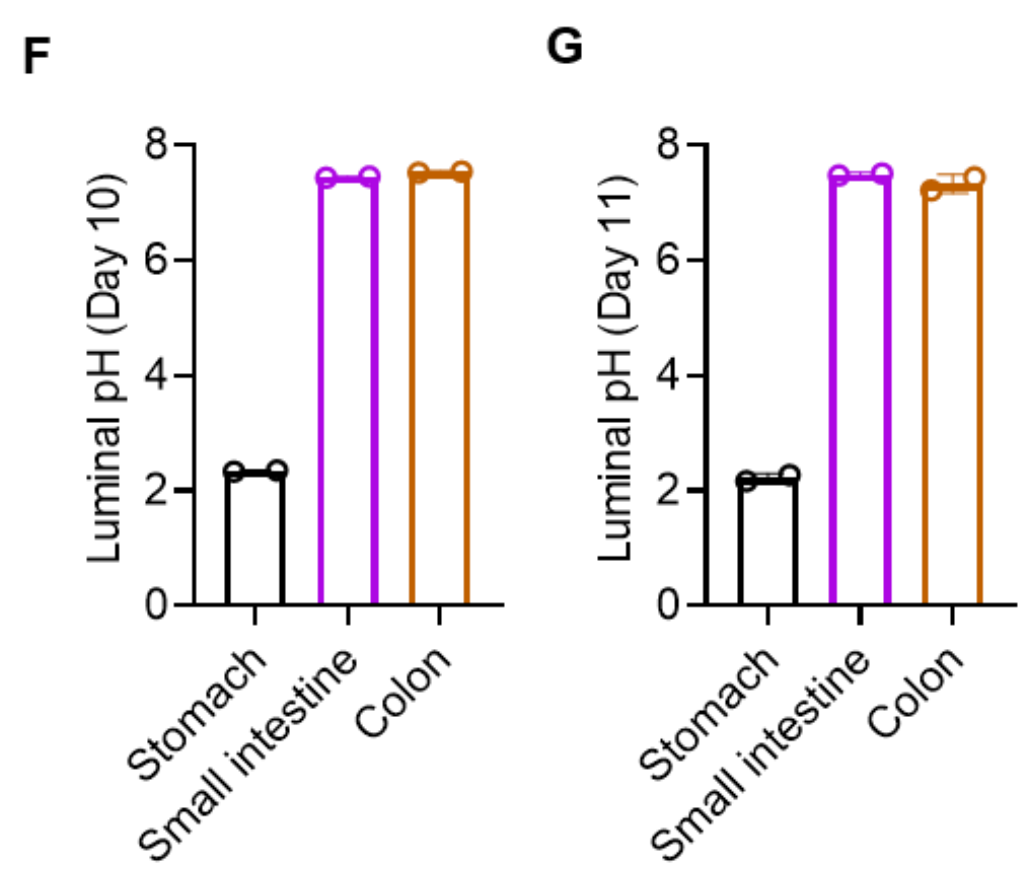

H

	Healthy mice	Colitis mice
Stomach	2.7	2.5
Small intestine	7.3	7.2
Colon	6.8	7.4

Figure S6. Measurement of luminal pH in the mice GI tract. A, Induction of acute colitis model and measurement of luminal pH in GI tract. **B,** Luminal pH in GI tract of healthy mice (n = 2). **C-G,** Luminal pH in GI tract of DSS-induced colitis mice at day 6 (**C**), day 8 (**D**), day 9 (**E**), day 10 (**F**), and day 11 (**G**) (n = 2). **H,** Average of luminal pH in the GI tract of healthy and colitis mice,

calculation based on the average pH value of all assessed mice for each group. Figure **S6A** was created with BioRender.com.

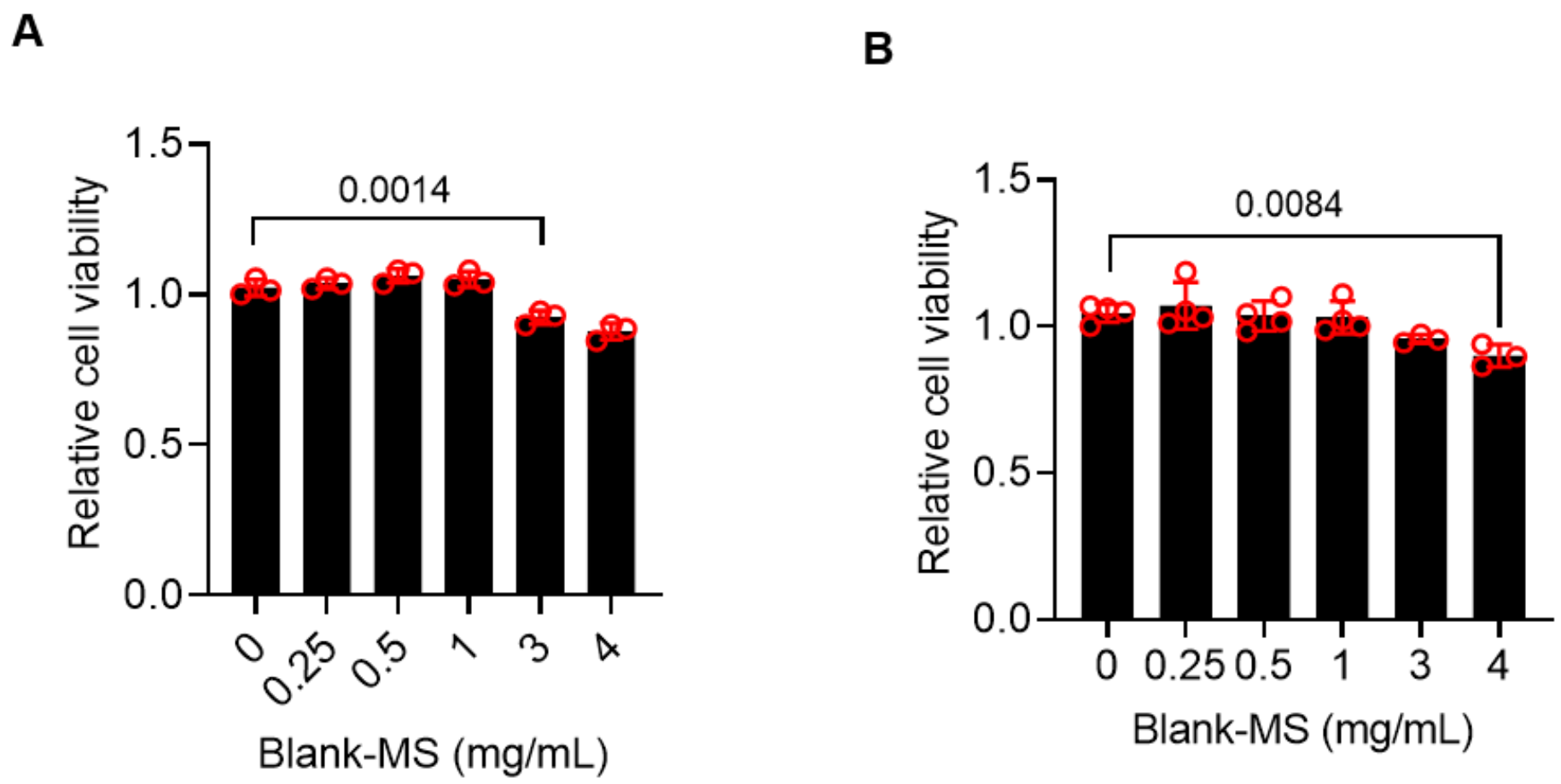

Figure S7. Cytotoxicity of Blank-MS. A, on Caco-2 cells (n = 3). **B,** on RAW 264.7 cells (n = 3-4). Data are presented as mean ± s.d. Data are analyzed using an unpaired two-tailed t-test (significant p-values are shown). Blank-MS indicating microsphere includes Eudragit FS 30 D and thioketal polymer without melatonin.

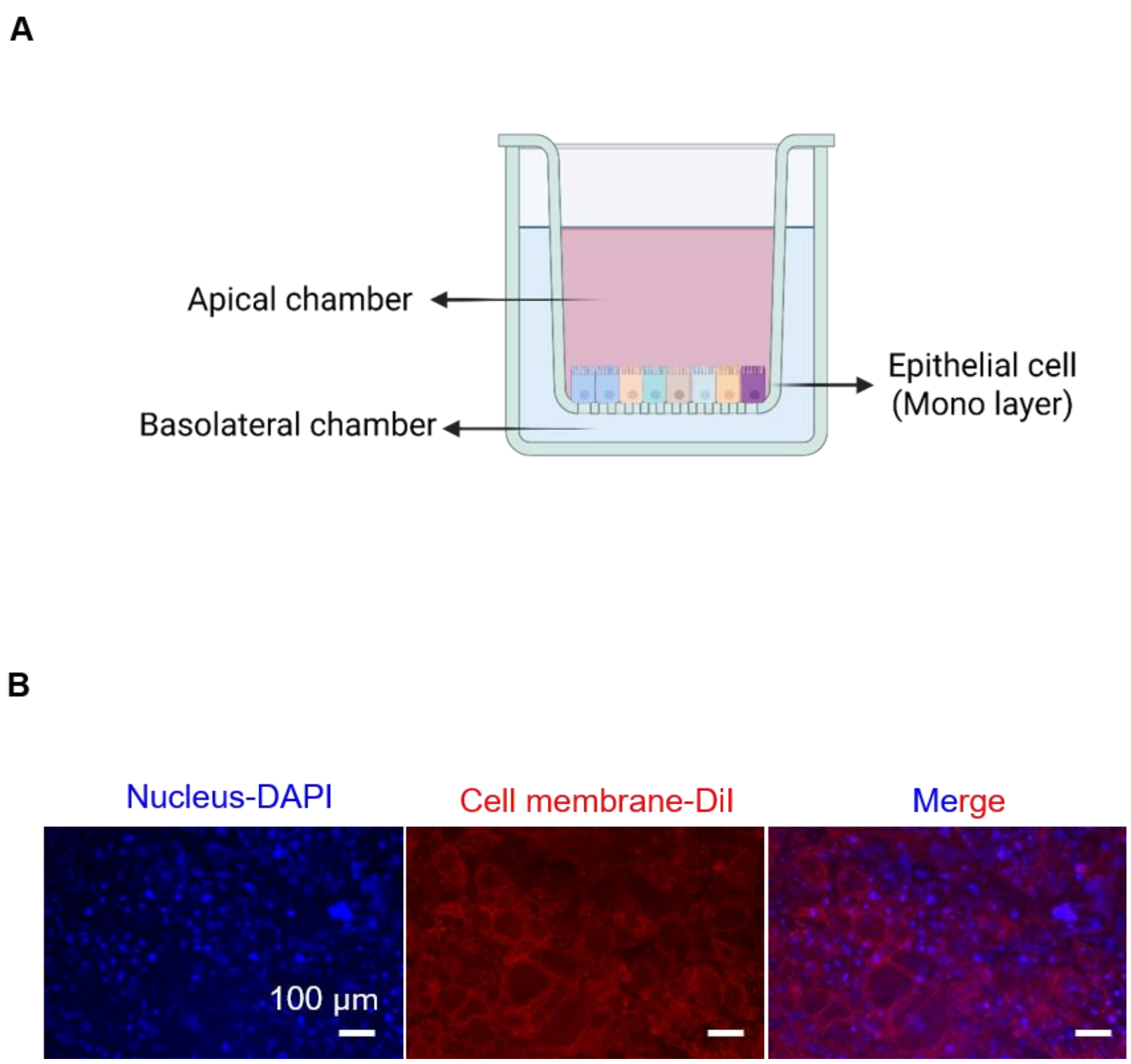

Figure S8. Caco-2 monolayer formation. A, Transwell model for Caco-2 layer. **B,** Fluorescence images of Caco-2 monolayer. Scale bar 100 µm. Figure **S8A** was created with BioRender.com.

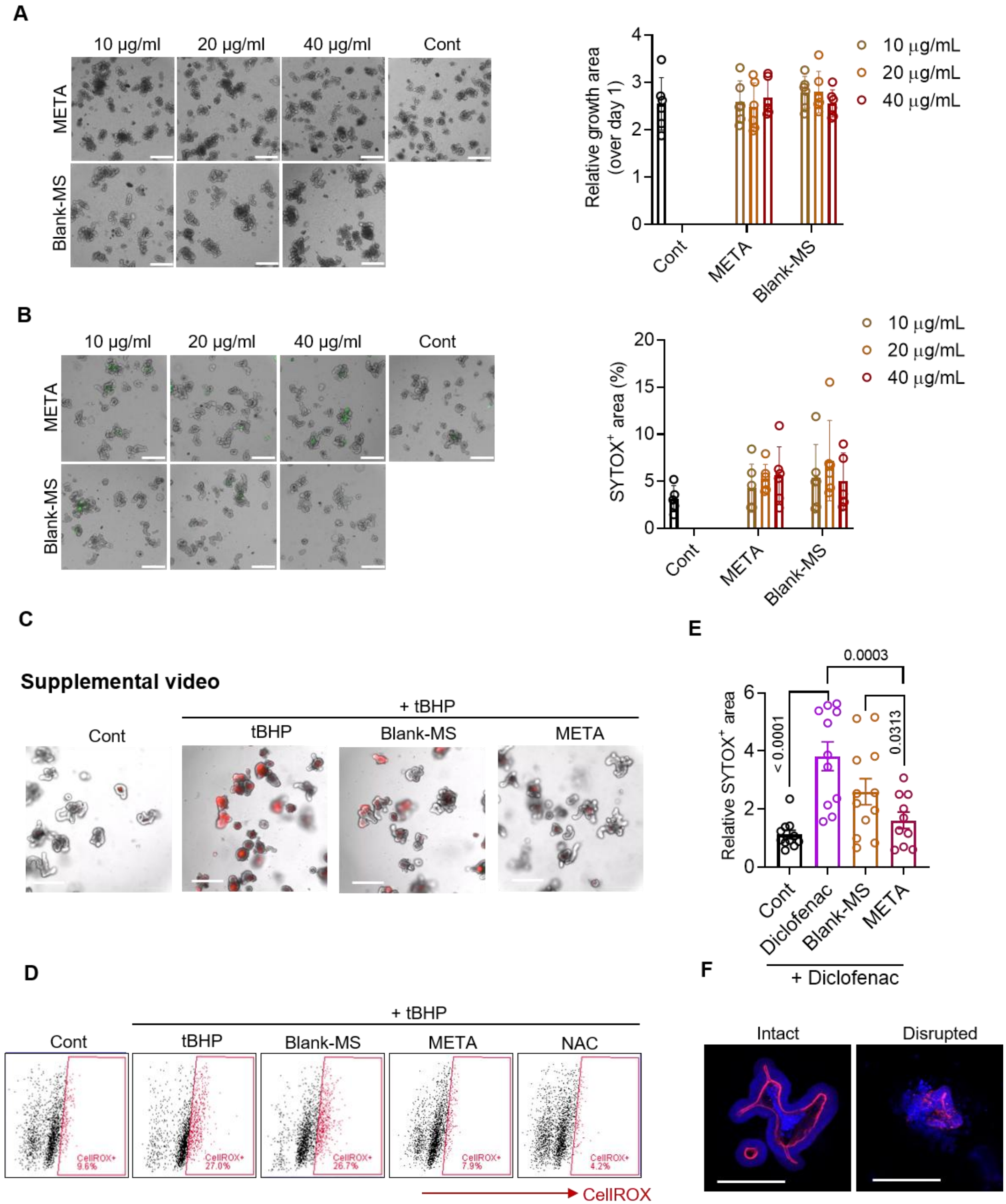

Figure S9. Impact of META on intestinal organoids (IOs). A, Images and relative growth area of IOs after treatment of META and Blank-MS (n = 6). Scale bar: 300 μm. **B,** Images and

percentage of $SYTOX^+$ area in IOs after treatment of META and Blank-MS (n = 5-6). Scale bar: 300 µm. **C,** Representative image of supplemental video of ROS levels in tBHP-induced IOs damage, scale bar: 300 µm. Please see the full movie in the supplemental video file for reference. **D,** Representative results of flow cytometric analysis of ROS-high cells in tBHP-induced IOs damage. N-Acetylcysteine (NAC) was included as a control. **E,** Relative $SYTOX^+$ area in diclofenac-induced IOs damage after treatments (n = 10-12). **F,** F-actin staining in healthy and diclofenac-disrupted IOs, scale bar: 300 µm. Data in (**A, B, E**) are presented as mean ± s.d. Data in (**E**) are analyzed using one-way ANOVA with the two-stage linear step-up method of Benjamini, Krieger, and Yekutieli (significant q values are shown).

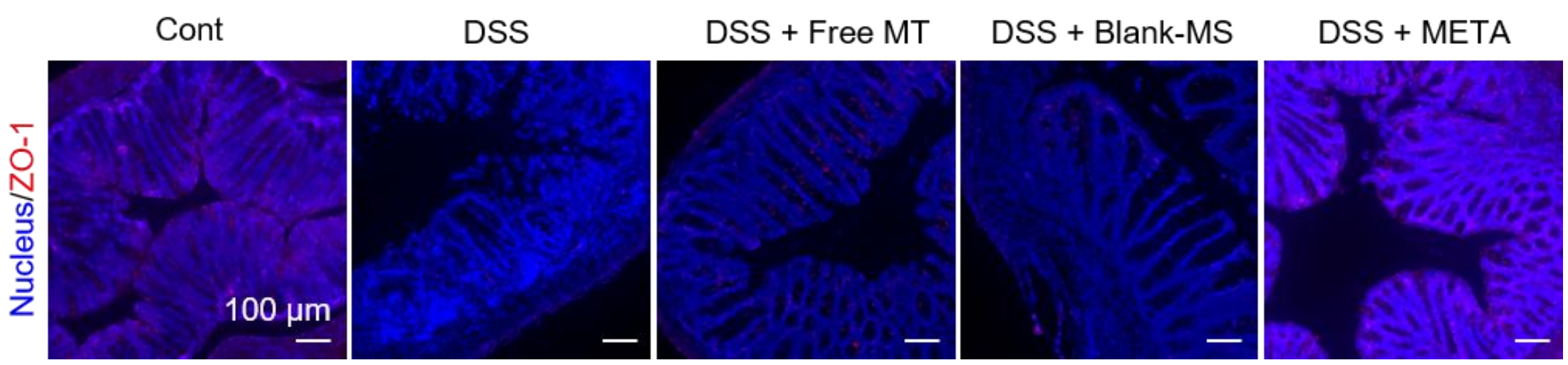

Figure S10. Immunofluorescence staining of ZO-1 protein in colon tissue. Scale bar: 100 μm.

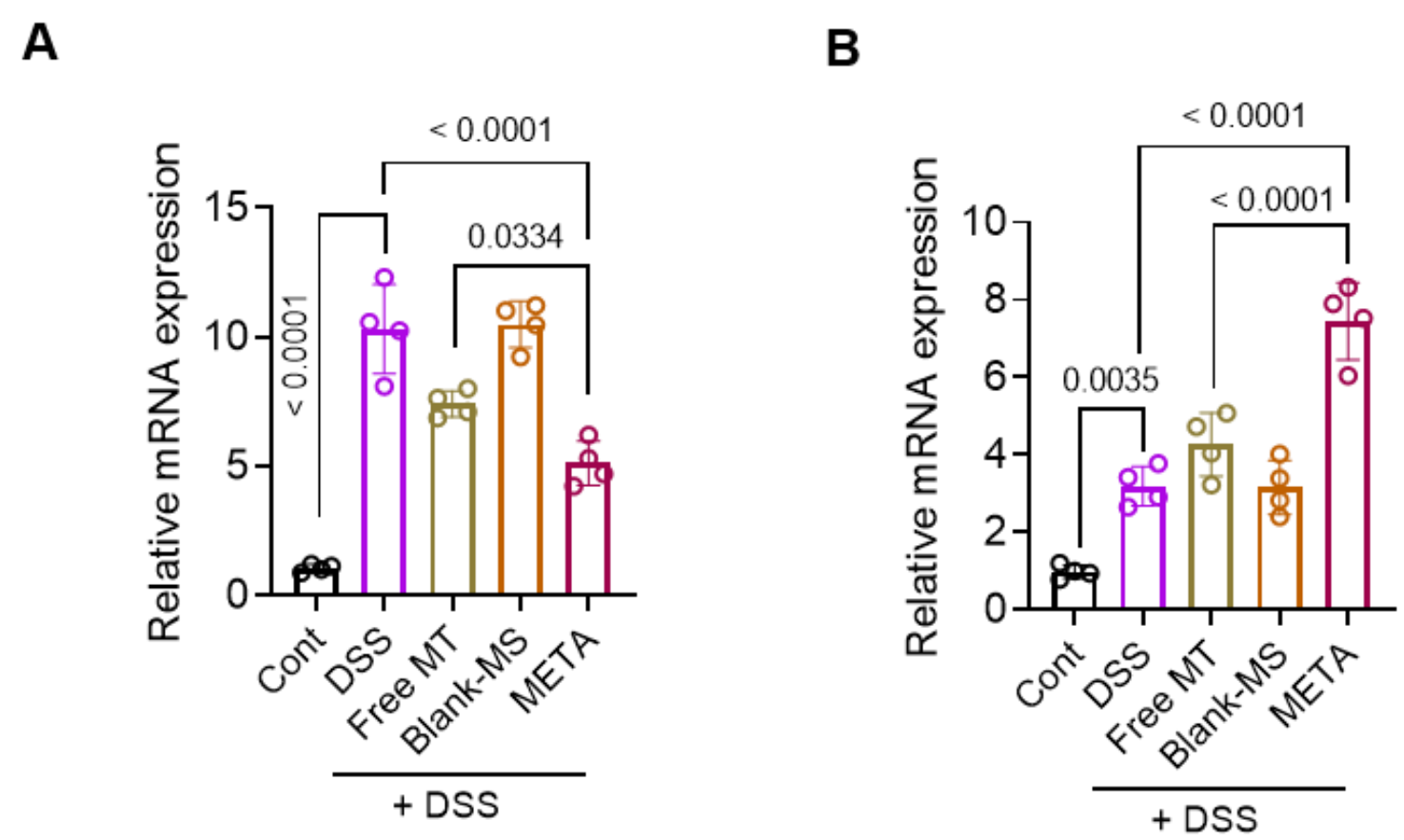

Figure S11. mRNA expression of CD86 and CD206 in the colonic mucosae. A, CD86 and **B,** CD206 (n = 4). Mononuclear cells were isolated from the colonic lamina propria and subjected to qRT-PCR analysis. Data are presented as mean ± s.d. Data are analyzed using one-way ANOVA with Tukey's multiple comparisons test (significant p values are shown).

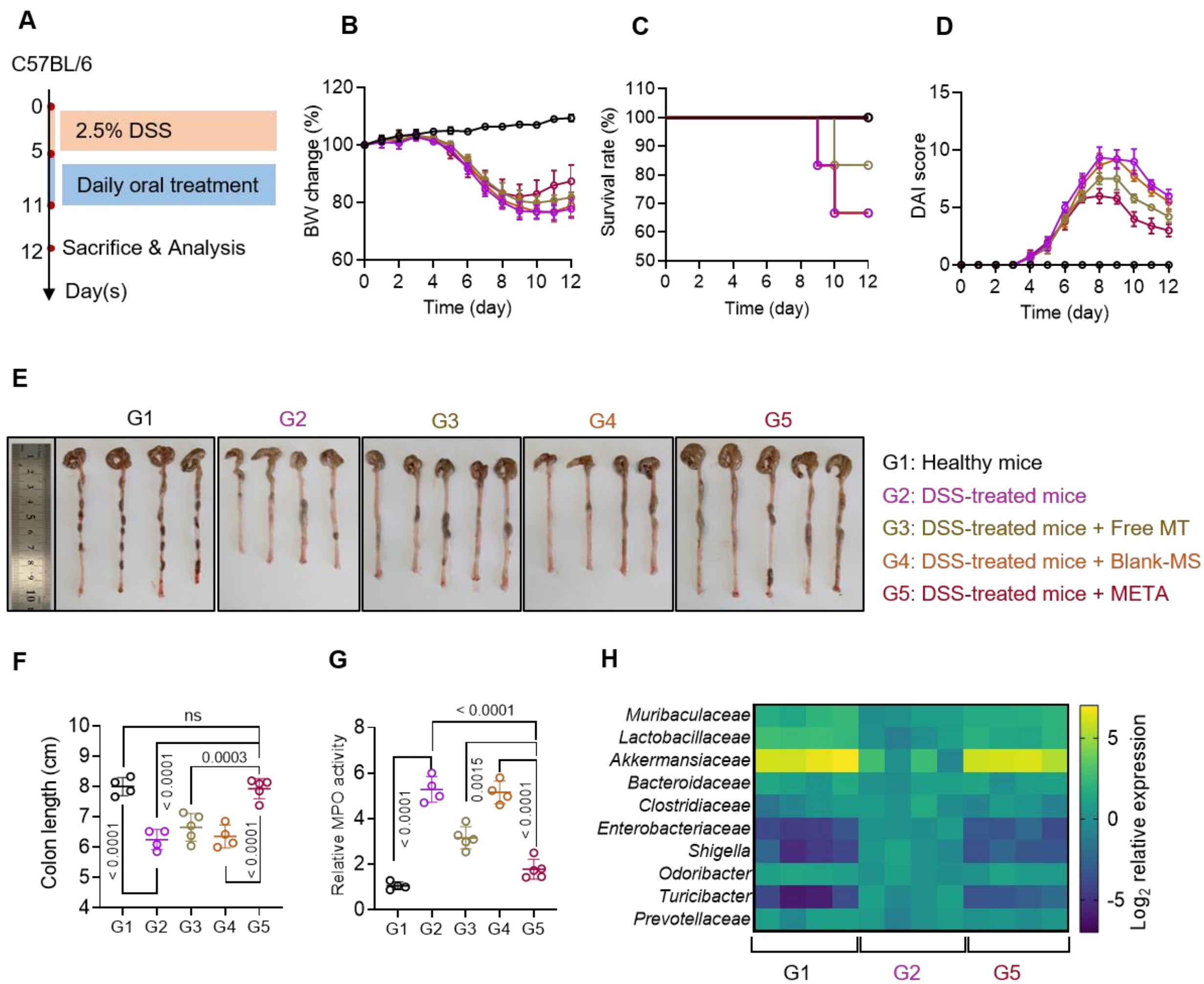

Figure S12. META effectively ameliorated UC. A, Induction of the mouse colitis model through oral DSS delivery and subsequent drug treatment. G1: Healthy mice, G2: DSS-treated mice, G3: DSS-treated mice + free melatonin (MT), G4: DSS-treated mice + Blank-MS, G5: DSS-treated mice + META. **B,** Body weight change measurement (n = 4-6 mice). **C,** Survival rate of mice (n = 4-6 mice). **D,** DAI score of mice (n = 4-6 mice). **E,** Images of colon tissues. **F,** Colon length measurement (n = 4-5 mice). **G,** Myeloperoxidase (MPO) activity in colon tissues analysis (n = 4-5 mice). **H,** Abundance of microbiota in the colon was quantified using qRT-PCR (n = 4 mice). Data in (**B, D, F, G**) are presented as mean ± s.d. Data in (**F, G**) are analyzed using one-way ANOVA with Tukey's multiple comparisons test (significant p values are shown).

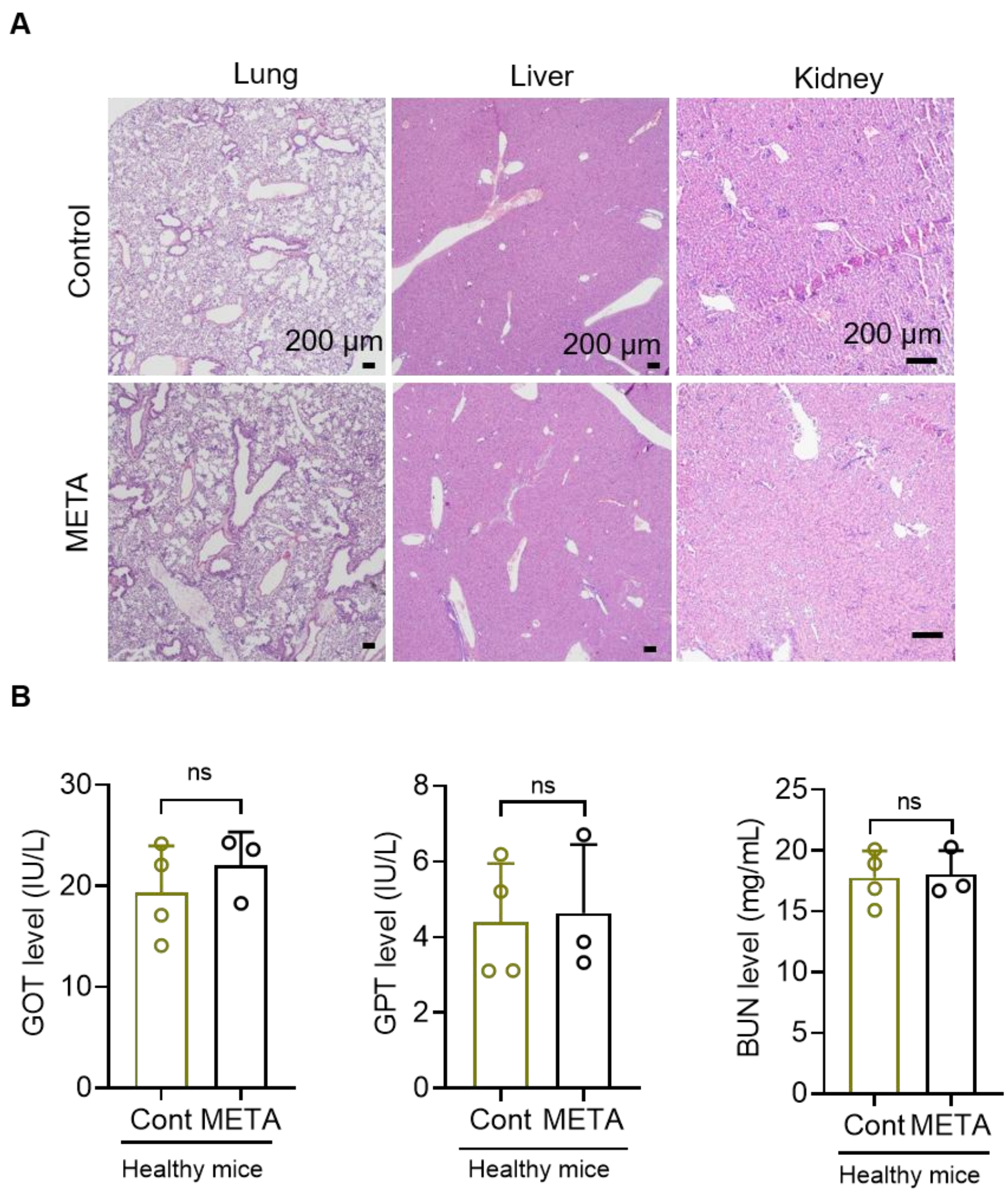

Figure S13. ***In vivo*** **safety of META. A,** Histological assessments (H&E) of major organs collected from mice after META treatment. Scale bar: 200 µm. **B,** Serum level quantification of biochemical index in mice, including GOT, GPT (liver function), and BUN (kidney function) (n = 3-4 mice). Data in (**B**) is presented as mean ± s.d. Data in (**B**) is analyzed using an unpaired two-tailed t-test. ns, no significance.

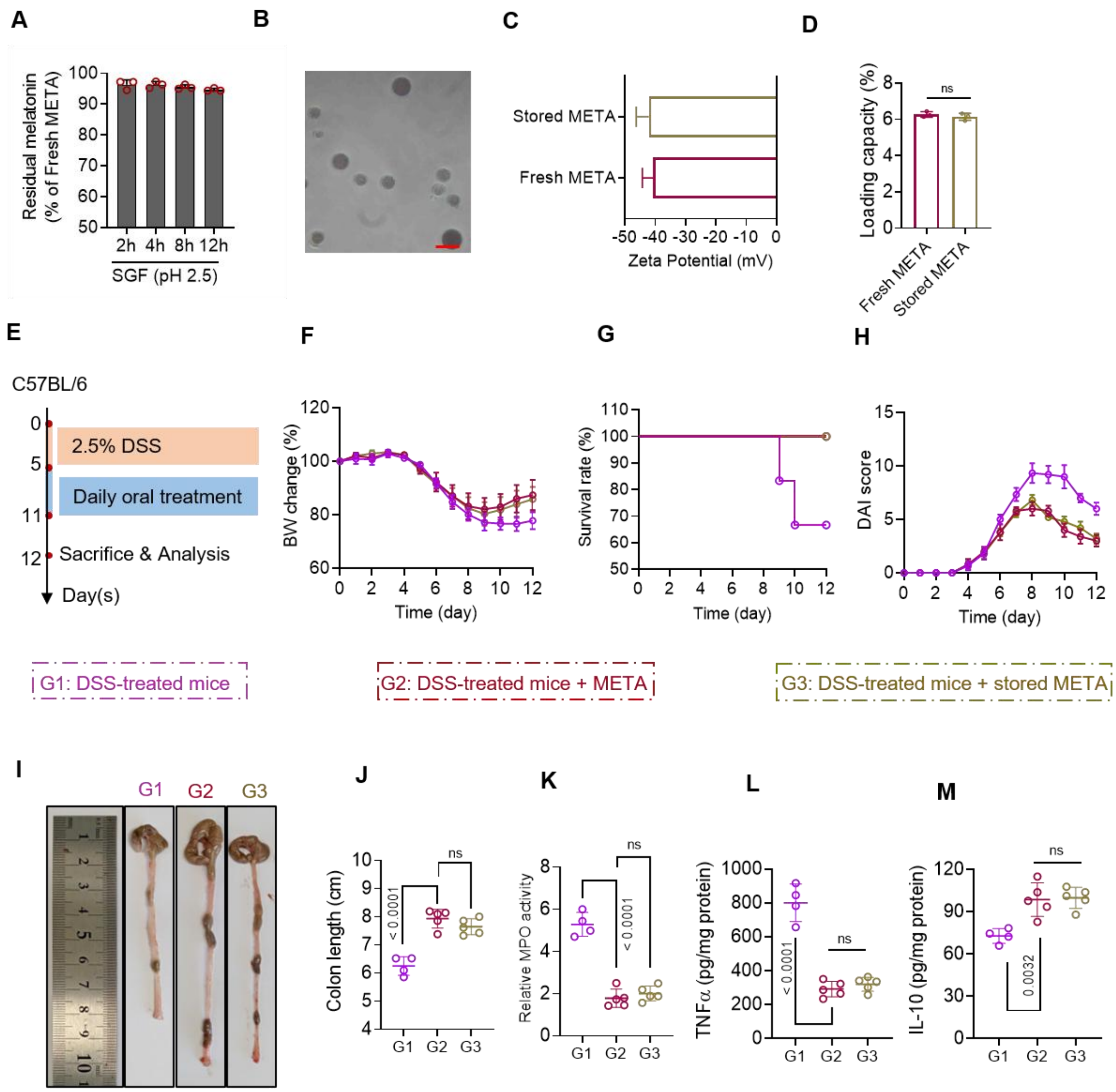

Figure S14. **The stability of META. A,** META stability under SGF conditions (n = 3). **B,** META morphology after 3-month storage at room temperature (RT). Scale bar: 5 µm. **C,** Zeta potential of META after 3-month storage at RT. **D,** Melatonin loading capacity of META after 3-month storage at RT. **E,** Induction of the mouse colitis model through oral DSS delivery and subsequent drug treatment. G1: DSS-treated mice, G2: DSS-treated mice + fresh META, G3: DSS-treated mice + stored META. **F,** Body weight change measurement (n = 5-6 mice). **G,** Survival rate of

mice (n = 5-6 mice). **H,** DAI score of mice (n = 5-6 mice). **I,** Representative images of colon tissues. **J,** Colon length measurement (n = 4-5 mice). **K,** Myeloperoxidase (MPO) activity in colon tissues analysis (n = 4-5 mice). **L-M,** ELISA measurement of colonic TNF-α (**L**) and IL-10 (**M**) (n = 4-5 mice). Data of **G1 (DSS-treated mice)** and **G2 (DSS-treated mice + META)** in this figure, including data in (**F, G, H, I, J, K**), were extracted from Figure **S12 (**Supplementary data) due to the same *in vivo* trial induction. Data are presented as mean ± s.d. Data are analyzed using one-way ANOVA with Tukey's multiple comparisons test (significant p values are shown).

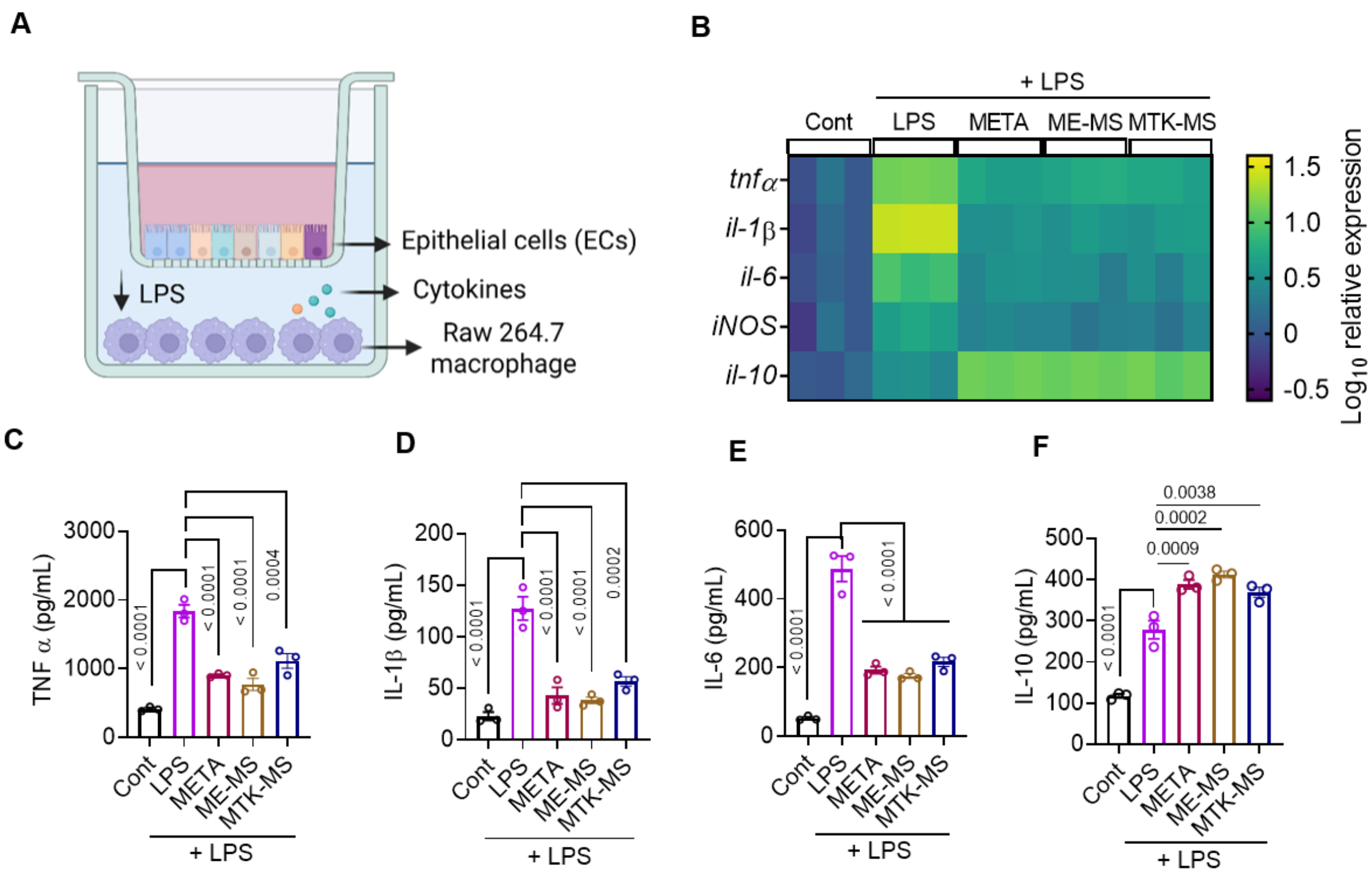

Figure S15. *In vitro* anti-inflammatory effects of individual polymer-based microspheres. A, Co-culture model of the epithelial Caco-2 cells (ECs) and RAW 264.7 macrophages. **B,** Expression of inflammatory and anti-inflammatory genes in RAW cells (n = 3). **C-F,** ELISA quantification of extracellular cytokines, including TNF-α, IL-1β, IL-6, and IL-10, respectively (n = 3). Data in (**C, D, E, F**) are presented as mean ± s.d. Data in (**C, D, E, F**) are analyzed using one-way ANOVA with Tukey's multiple comparisons test (significant p values are shown). Figure **S15A** was created with BioRender.com.

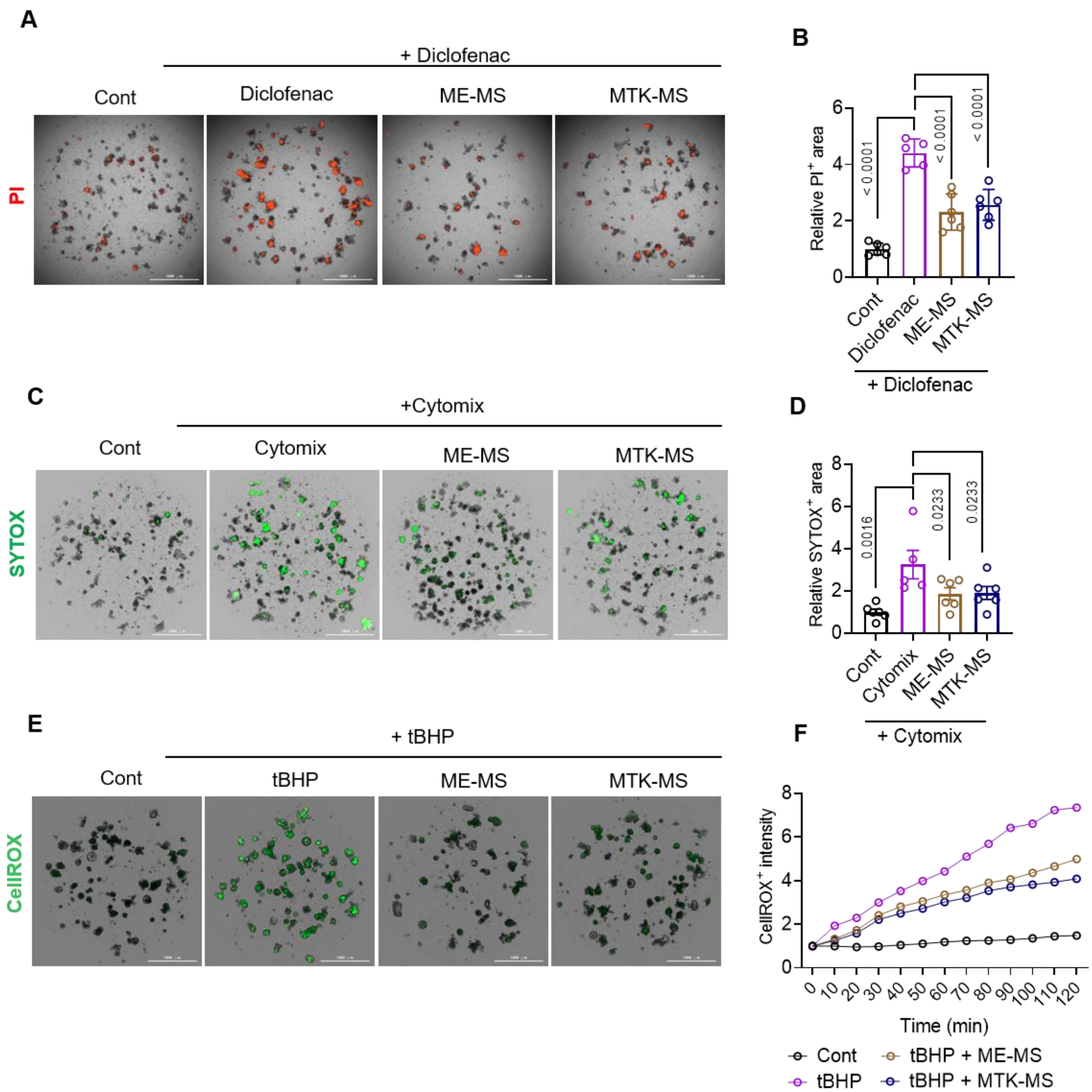

Figure S16. Protective effects of individual polymer-based microspheres on intestinal organoids. A, Propidium iodide (PI) staining depicts diclofenac-induced IO damage. Scale bar: 1000 μm. **B,** PI-positive area of IO measurement (n = 5-6). **C,** Cytomix-induced IOs damage stained with SYTOX. Scale bar: 1000 μm. **D,** SYTOX-positive area of IO measurement (n = 5-6). **E,** CellROX staining showing oxidative stress-induced damage in IOs treated with tBHP. Scale bar: 1000 μm. **F,** Time course of CellROX intensity in IOs. Data in (**B, D**) are presented as mean

± s.d. Data in (**B, D**) are analyzed using one-way ANOVA with Tukey's multiple comparisons test (significant p values are shown).

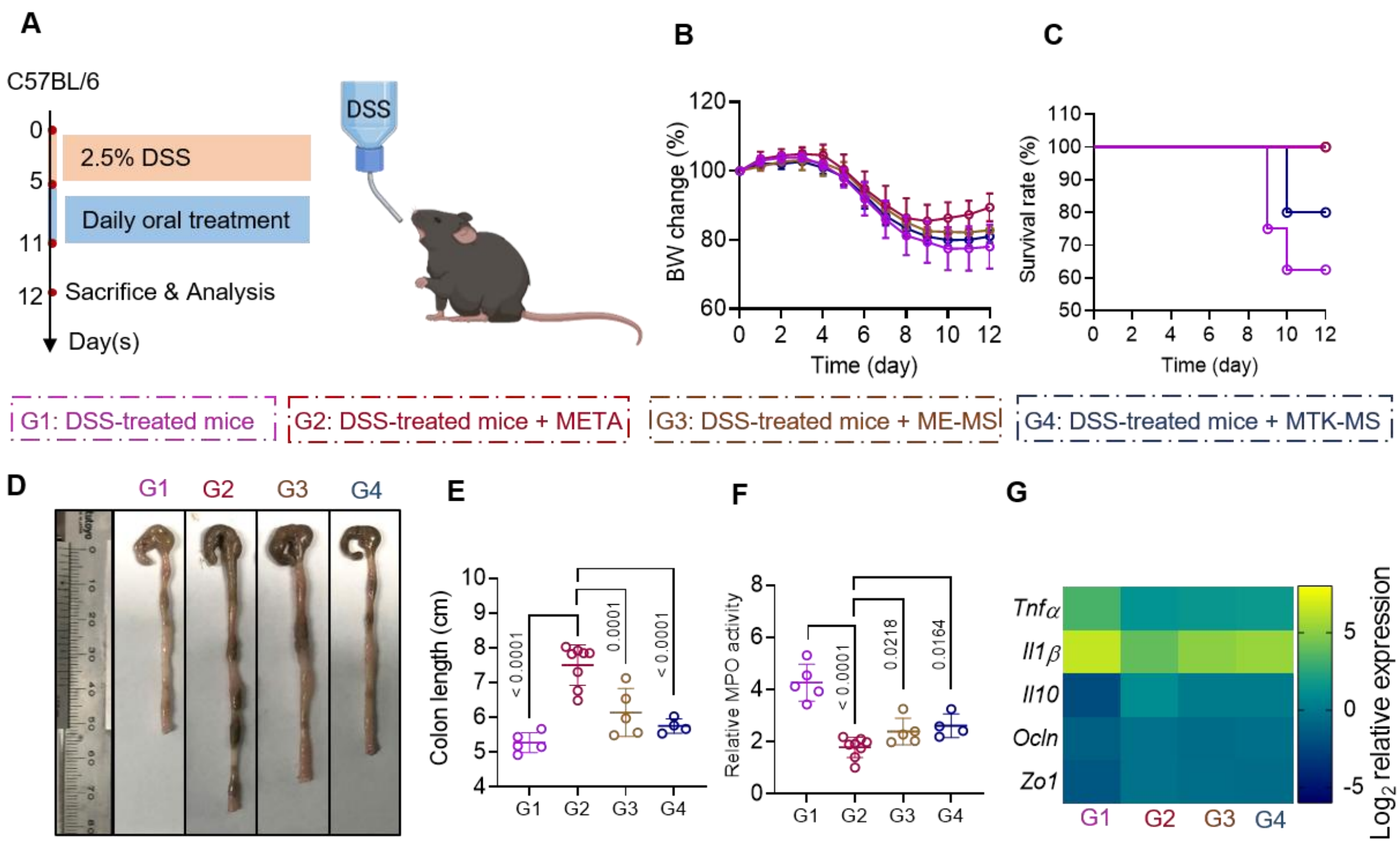

Figure S17. Superior therapeutic effects of META on DSS-induced colitis mice. A, Induction of the mouse colitis model through oral DSS delivery and subsequent drug treatment. G1: DSS-treated mice, G2: DSS-treated mice + META, G3: DSS-treated mice + ME-MS, G4: DSS-treated mice + MTK-MS. **B,** Body weight change measurement (n = 4-8 mice). **C,** Survival rate of mice (n = 5-8 mice). **D,** Representative images of colon tissues. **E,** Colon length measurement (n = 4-8 mice). **F,** Myeloperoxidase (MPO) activity in colon tissues analysis (n = 4-8 mice). **G,** Heat map of gene expression in colon tissues (n = 3-5 mice). Data of **G1 (DSS-treated mice)** and **G2 (DSS-treated mice + META)** in this figure, including in (**B, C, E, F, G**) were extracted from the main Figure **7** due to the same *in vivo* trial induction. Data are presented as mean ± s.d. Data are analyzed using one-way ANOVA with Tukey's multiple comparisons test (significant p values are shown). Figure **S17A** was created with BioRender.com.

Supplementary tables

Table S1. Clinical trials of oral melatonin for ulcerative colitis (2005-2025)

Trial	Year	Population	Treatment	Dosage form	Reference
Shahrokh et al.	2021	Mild-moderate UC (n=30)	3 mg/day oral for 3 months + standard therapy	Free melatonin	[5]
Chojnacki et al.	2011	Patients with left-sided UC (n=30)	Oral melatonin (5 mg/day) with mesalazine	Free melatonin	[6]
NCT00790478 Pilot	2008-2012	Mild-moderate UC	Oral melatonin as an adjunctive	Free melatonin	ClinicalTrials.gov

Only 3 clinical trials were identified (all oral free melatonin, no modified delivery systems). Doses: 3-5 mg/day (standard human dose). All adjunctive therapy with standard UC treatments. No nanoparticle/microsphere melatonin clinical trials in UC patients have been found.

Table S2. Formulations used in this study

Objectives	Formulations
Loading capacity (LC%)	$LC\% = \frac{\text{Amount of drug loaded in MS}}{\text{Actual amount of MS}} \times 100\%$
Entrapment efficiency (EE%)	$EE\% = \frac{\text{Amount of drug loaded in MS}}{\text{Initial amount of drug added}} \times 100\%$
Similarity factor f_2	$f_2 = 50 \times \log\left\{\left[1 + \frac{1}{n}\sum_{t=1}^{n}(R_t - T_t)^2\right]^{-0.5} \times 100\right\}$
Transepithelial electrical resistance (TEER)	$TEER = (R_m - R_i) \times A\ (ohm.cm^2)$

where n is the number of time points, R_t and T_t are the dissolution values of different formulations at time point t, respectively. R_m is the resistance of the transmembrane, R_i is the intrinsic resistance of the cell medium, and A is the Transwell membrane surface area. MS: Microsphere

Table S3. Disease activity index criteria

**Parameters**	**Criteria**	**Score**
Weight loss	0-1%	0
	1-5%	1
	5-10%	2
	10-20%	3
	> 20%	4
Rectal bleeding	Negative	0
	Minor - moderate	2
	Severe	4
Stool consistency	Hard	0
	Soft	2
	Diarrhea	4

Table S4. Bacterial primer sequences used in RT-PCR analysis

Bacterial genera	Primer	Nucleotide sequence
Total bacteria	F	5'-AGAGTTTGATCATGGCTCAG-3'
	R	5'-ACCGCGACTGCTGCTGGCAC-3'
Muribaculaceae	F	5'-AGGCCCGGTTCATACAGCTC-3'
	R	5'-GCGAATCCAAGTGCTGTCGG-3'
Lactobacillaceae	F	5'-AGCAGTAGGGAATCTTCCA-3'
	R	5'-CACCGCTACACATGGAG-3'
Akkermansiaceae	F	5'-CAGCACGTGAAGGTGGGGAC-3'
	R	5'-CCTTGCGGTTGGCTTCAGAT-3'
Bacteroidaceae	F	5'-GGTTCTGAGAGGAGGTCCC-3'
	R	5'-GCTGCCTCCCGTAGGAGT-3'
Clostridiaceae	F	5'-AAATGACGGTACCTGACTAA-3'
	R	5'-CTTTGAGTTTCATTCTTGCGAA-3'
Enterobacteriaceae	F	5'-GTGCCAGCAGCCGCGGTAA-3'
	R	5'-GCCTCAAGGGCACAACCTCCAAG-3'
Shigella	F	5'-CAAGTCCGTAAATTCATTCTCTCTTT-3'
	R	5'-GCTGGAAAAACTCAGTGCCTCCC-3'
Odoribacter	F	5'-ATGTAATGATGAGCACTCTAACGG-3'
	R	5'-GGCTTTTGAGATTGGCATCC-3'
Turicibacter	F	5'-ACGGGGACAACGATTGGAAA-3'
	R	5'-AGTGATGCCAGGAGCATCTT-3'
Prevotellaceae	F	5'-GGTGTCGGCTTAAGTGCCAT-3'
	R	5'-CGGACGTAAGGGCCGTGC-3'

Table S5. Primer sequences used in RT-PCR analysis

Target gene	Species	Primer	Nucleotide sequence
Actb	Mouse	F	5'-CAT TGC TGA CAG GAT GCA GAA GG-3'
		R	5'-TGC TGG AAG GTG GAC AGT GAG G-3'
Gapdh	Mouse	F	5'-ACC ACA GTC CAT GCC ATC AC-3'
		R	5'-TCC ACC ACC CTG TTG CTG TA-3'
Aanat	Mouse	F	5'-CCA GTG CGT TTG AGA TTG AGC G-3'
		R	5'-AAG GCA GCC TTC CTC AAA CCA G-3'
Hiomt	Mouse	F	5'-GCA GCC TCC TGC TCT ACC TG-3'
		R	5'-ACC TGT AGA TGG CGG TGA AGG-3'
Tph1	Mouse	F	5'-TGT TGA CTG CGA CAT CAG CCG A-3'
		R	5'-GGA AAC CAA GGG ACA GTC TCC A-3'
Tnfα	Mouse	F	5'-GGT GCC TAT GTC TCA GCC TCT T-3'
		R	5'-GCC ATA GAA CTG ATG AGA GGG AG-3'
Il1β	Mouse	F	5'-TGG ACC TTC CAG GAT GAG GAC A-3'
		R	5'-GTT CAT CTC GGA GCC TGT AGT G-3'
P65	Mouse	F	5'-TCC TGT TCG AGT CTC CAT GCA G-3'
		R	5'-GGT CTCA TAG GTC CTT TTG CGC-3'
Cox2	Mouse	F	5'-GCG ACAT ACT CAA GCA GGA GCA-3'
		R	5'-AGT GGT AAC CGC TCA GGT GTT G-3'
Il6	Mouse	F	5'-TAC CAC TTC ACA AGT CGG AGG C-3'
		R	5'-CTG CAA GTG CAT CAT CGT TGT TC-3'
Il10	Mouse	F	5'-CGG GAA GAC AAT AAC TGC ACC C-3'
		R	5'-CGG TTA GCA GTA TGT TGT CCA GC-3'
iNOS	Mouse	F	5'-GAG ACA GGG AAG TCT GAA GCA C-3'
		R	5'-CCA GCA GTA GTT GCT CCT CTT C-3'
Tgfβ	Mouse	F	5'-TGA TAC GCC TGA GTG GCT GTC T-3'
		R	5'-CAC AAG AGC AGT GAG CGC TGA A-3'
Ocln	Mouse	F	5'-TGG CAA GCG ATC ATA CCC AGA G-3'
		R	5'-CTG CCT GAA GTC ATC CAC ACT C-3'
Zo1	Mouse	F	5'-GTT GGT ACG GTG CCC TGA AAG A-3'
		R	5'-GCT GAC AGG TAG GAC AGA CGA T-3'
Ecad1	Mouse	F	5'-GGT CAT CAG TGT GCT CAC CTC T-3'
		R	5'-GCT GTT GTG CTC AAG CCT TCA C-3'
Emr1	Mouse	F	5'-CGT GTT GTT GGT GGC ACT GTG A-3'
		R	5'-CCA CATC AGT TGT TCC AGG AGA C-3'
Stat3	Mouse	F	5'-AGG AGT CTA ACA ACG GGC AGC CT-3'
		R	5'-GTG GTA CAC CTC AGT CTC GAA G-3'
Gapdh	Human	F	5'-GTC GGA GTC AAC GGA TTT GG-3'
		R	5'-GGG TGG AAT CAT TGG AAC AT-3'
Nrf2	Human	F	5'-CAC ATC CAG TCA GAA ACC AGT GG-3'
		R	5'-GGA ATG TCT GCG CCA AAA GCT G-3'
Ho1	Human	F	5'-CCA GGC AGA GAA TGC TGA GTT C-3'

		R	5’-AAG ACT GGG CTC TCC TTG TTG C-3’
Ocln	Human	F	5’-ATG GCA AAG TGA ATG ACA AGC GG-3’
		R	5’-CTG TAA CGA GGC TGC CTG AAG T-3’
Cldn4	Human	F	5’-AGT GCA AGG TGT ACG ACT CGC T-3’
		R	5’-CGC TTT CAT CCT CCA GGC AGT T-3’
Zo1	Human	F	5’-GTC CAG AAT CTC GGA AAA GTG CC-3’
		R	5’-CTT TCA GCG CAC CAT ACC AAC C-3’
Ecad1	Human	F	5’-GCC TCC TGA AAA GAG AGT GGA AG-3’
		R	5’-TGG CAG TGT CTC TCC AAA TCC G-3’

References

1. Chen Q, Wang H, Liu Y, Song Y, Lai L, Han Q, et al. Inducible microRNA-223 down-regulation promotes TLR-triggered IL-6 and IL-1β production in macrophages by targeting STAT3. PLoS One. 2012; 7: e42971.

2. Arras LD, Guthrie BS, Alper S. Using RNA-interference to investigate the innate immune response in mouse macrophages. J Vis Exp. 2014; 93(93): e51306.

3. Regmi S, Pathak S, Nepal MR, Shrestha P, Park J, Kim JO, et al. Inflammation-triggered local drug release ameliorates colitis by inhibiting dendritic cell migration and Th1/Th17 differentiation. J Control Release. 2019; 316: 138-49.

4. Shimizu K, Seiki I, Goto Y, Murata T. Measurement of the intestinal pH in mice under various conditions reveals alkalization induced by antibiotics. Antibiotics. 2021; 10: 180.

5. Shahrokh S, Qobadighadikolaei R, Abbasinazari M, Haghazali M, Aghdaei HA, Abdi S, et al. Efficacy and safety of melatonin as an adjunctive therapy on clinical, biochemical, and quality of life in patients with ulcerative colitis. Iran J Pharm Res. 2021; 20: 197-205.

6. Chojnacki C, Wisniewska-Jarosinska M, Walecka-Kapica E, Klupinska G, Jaworek J, Chojnacki J. Evaluation of melatonin effectiveness in the adjuvant treatment of ulcerative colitis. J Physiol Pharmacol. 2011; 62: 327-34.
